# Rhizosphere Bacterial Communities Differ According to Fertilizer Regimes and Cabbage (*Brassica oleracea* var. *capitata* L.) Harvest Time, but Not Aphid Herbivory

**DOI:** 10.3389/fmicb.2018.01620

**Published:** 2018-07-23

**Authors:** Flora J. M. O’Brien, Marc G. Dumont, Jeremy S. Webb, Guy M. Poppy

**Affiliations:** ^1^Biological Sciences, University of Southampton, Southampton, United Kingdom; ^2^NIAB EMR, East Malling, United Kingdom

**Keywords:** rhizosphere, fertilizers, plant growth, bacterial communities, *Brassica oleracea*

## Abstract

Rhizosphere microbial communities are known to be highly diverse and strongly dependent on various attributes of the host plant, such as species, nutritional status, and growth stage. High-throughput 16S rRNA gene amplicon sequencing has been used to characterize the rhizosphere bacterial community of many important crop species, but this is the first study to date to characterize the bacterial and archaeal community of *Brassica oleracea* var. *capitata*. The study also tested the response of the bacterial community to fertilizer type (organic or synthetic) and N dosage (high or low), in addition to plant age (9 or 12 weeks) and aphid (*Myzus persicae*) herbivory (present/absent). The impact of aboveground herbivory on belowground microbial communities has received little attention in the literature, and since the type (organic or mineral) and amount of fertilizer applications are known to affect *M. percicae* populations, these treatments were applied at agricultural rates to test for synergistic effects on the soil bacterial community. Fertilizer type and plant growth were found to result in significantly different rhizosphere bacterial communities, while there was no effect of aphid herbivory. Several operational taxonomic units were identified as varying significantly in abundance between the treatment groups and age cohorts. These included members of the S-oxidizing genus *Thiobacillus*, which was significantly more abundant in organically fertilized 12-week-old cabbages, and the N-fixing cyanobacteria *Phormidium*, which appeared to decline in synthetically fertilized soils relative to controls. These responses may be an effect of accumulating root-derived glucosinolates in the *B. oleracea* rhizosphere and increased N-availability, respectively.

## Introduction

Healthy soils are vital for agricultural production and, therefore, an important pillar for achieving food security. Many soil microorganisms perform an array of valuable ecosystem services, which may enhance plant growth, such as nutrient cycling, carbon storage, and soil remediation (Daily et al., 1997), while others are pathogenic toward plants and can cause reductions in crop yields (Hayward, 1991; Navas-Cortés et al., 2000; Paulitz et al., 2002). Thus, compositional shifts in these communities can have significant consequences for the nutritional status and health of crops. Modern farming practices, such as fertilizer applications, can alter soil microbial communities through their impact on various edaphic factors, including soil moisture, pH (Li et al., 2012; Tripathi et al., 2012; Blasiak et al., 2014), nutrient availability, organic matter content, and temperature (Kowalchuk and Stephen, 2001; Bulluck et al., 2002; Bates et al., 2011; de Vries et al., 2012b). In comparison to mineral fertilizers, organic fertilizers (e.g., animal manures and compost) have been reported to enhance the bacterial richness (number of species) and lower evenness (relative abundance of taxa) of soil communities (Hartmann et al., 2015; Lupatini et al., 2017). Evenness of soil bacterial communities has been associated with functional stability and the resilience to environmental disturbance (Wittebolle et al., 2009), whereas increased species richness has been linked to greater activity (respiration) and resource-use efficiency of microbial communities (Bell et al., 2005; Saleem et al., 2016). However, the relationship between soil microbial communities and fertilizer inputs is complex, and some studies have found no difference in bacterial richness or evenness between the two management systems (Pershina et al., 2015). The amount of nitrogen (N) applied to soils has also been reported to impact microbial communities (Nemergut et al., 2008; Ramirez et al., 2010, 2012; Fierer et al., 2012), leading to a reduction in soil microbial diversity in some cases (Li et al., 2016).

Plants can substantially alter soil microbial communities through the production of carbon-rich root exudates (rhizodeposition), which include various plant hormones (Cardoso et al., 2014), organic acids, amino acids (de Weert et al., 2002; Carvalhais et al., 2011), phenolic compounds (Badri et al., 2013a), phytosiderophores (iron-chelators) (Takagi et al., 1984), mucilage, and sloughed-off root cap cells (Iijima et al., 2000). Consequently, the area of soil immediately surrounding the roots, known as the rhizosphere, can exhibit significantly higher microbial abundances than the bulk (root-free) soil. Bulk soil tends to be dominated by oligotrophic (slow-growing) bacteria, whereas copiotrophic (fast-growing) bacteria are more typical of plant rhizosphere communities (Maloney et al., 1997; Dennis et al., 2010; Uksa et al., 2015; Ridl et al., 2016). Rhizodeposition rates vary with plant species and age, as well as environmental conditions (Nguyen, 2003; Jones et al., 2004). Research indicates that plants can use these exudates to actively recruit “plant growth promoting rhizobacteria” (PGPR) that may enhance plant growth either directly, for example, by increasing nutrient availability, or indirectly by stimulating plant defenses against pests or pathogens (Conrath et al., 2002).

*Brassica* plants include many economically important crop species, such as broccoli, kale, mustard, and cabbage. A characteristic trait of brassicas is the production of sulfur-rich secondary metabolites called glucosinolates. These compounds are valued both for their role in plant defense against insect herbivory and for their health benefits, which include anti-carcinogenic properties (Kiddle et al., 2001; Mazzola et al., 2001). *Brassica* plants can also be used as a natural soil fumigant as glucosinolates have been shown to have antifungal and antibacterial effects (Mithen et al., 1986; Tierens et al., 2001; Aires et al., 2009). Since glucosinolates are produced in all plant organs, it may be expected that the roots of Brassicaceous plants may significantly alter the surrounding soil microbial community. This has been demonstrated to an extent by Bressan et al. (2009) who used DGGE fingerprinting to show that the glucosinolate profile of *Arabidopsis thaliana* roots influenced the bacterial and fungal community of rhizospheric soil. Similarly, Hanschen et al. (2015) found that the addition of *Brassica*-derived glucosinolates significantly altered the soil bacterial community and that nitriles were a major degradation product, which may be indicative of lactic acid bacteria activity (Mullaney et al., 2013).

Soil-plant-insect interactions are influenced by top–down or bottom–up forces. *Top–down* forces are regulated by their consumers (i.e., plant growth being regulated by herbivorous insects), whereas *bottom–up* forces are determined by resource quantity and quality (Wardle et al., 2004). Rhizosphere microbes can influence aboveground herbivory by affecting the nutrient status (quality) and biomass (quantity) of the host plant through their influence on soil nutrient availability and pathogen status, thus acting as a bottom–up force (Borowicz, 1997; Badri et al., 2013b). For instance, root colonization of barrelclover (*Medicago truncatula* Gaertn.) by the mycorrhizal fungus *Glomus versiforme* has been shown to affect the metabolic profile of the plant, which can have important implications in herbivory defense (Harrison and Dixon, 1993). Likewise, aboveground herbivory of a host plant can influence the microbial rhizosphere community via herbivory-induced changes in plant metabolism and root exudation. Artificial foliar herbivory (defoliation by clipping) of the grass *Poa pratensis* L. has been reported to result in increased photosynthetic and root C exudation rates, which resulted in increased soil microbial activity and, consequently, enhanced N availability (Hamilton and Frank, 2001). However, the accuracy of artificial defoliation in representing actual herbivory has been discredited in other studies (Baldwin, 1988; Frost and Hunter, 2004). Most studies of above-ground herbivory effects on soil microbial communities have focused on grazing animals (Wang et al., 2006) or leaf chewers (Frost and Hunter, 2004), whereas rhizosphere community responses to phloem-feeding insects, such as aphids, are poorly understood. However, studies have indicated that plants subjected to aphid herbivory are less susceptible to soil-borne pathogens and recruit higher numbers of beneficial rhizobacteria (Yang et al., 2011; Lee et al., 2012).

In this study, we sequenced 16S rRNA amplicons using Illumina MiSeq to assess the soil bacterial and archaeal community of an agricultural soil at various stages during the pot cultivation of Derby Day cabbages (*Brassica oleracea* var. *capitata* L.). The objective of this study was to determine the response of the soil rhizosphere community to (i) *B. oleracea* age at harvest, (ii) organic and synthetic fertilizers at two N doses, and (iii) the effect of herbivory by the green peach-potato aphid (*Myzus persicae*). Based on these groupings, this study aimed to answer three key questions:

(1) Does the alpha diversity (i.e., diversity within samples) of the soil microbial communities differ between groups?
(2) Is the beta diversity (i.e., the presence/absence and abundance of taxa) of the soil communities distinct between groups?
(3) What are the main bacterial taxa responsible for these differences, if any, in beta diversity?

## Materials and Methods

### Soil Collection

The soil used in this study was collected from an agricultural field in Ipsden, South Oxfordshire (51°32′59.559″ N, 1°05′8.43″ W) in March 2013. The soil from this field site has been characterized previously in de Vries et al. (2012a). Soil samples were taken from the top 15 cm of the soil horizon and were stored at 4°C upon arrival at the University of Southampton until further processing. The soil was air-dried and sieved (<2 mm), before being used to fill 4″-diameter plant pots (approximately 400 g pot^-1^). Soil nutrient analysis (total C and N) was performed by NRM Laboratories Ltd (Berkshire, United Kingdom), and pH was determined using the slurry technique (soil:distilled water 1:5).

### Fertilizer Treatments

Fertilizers were applied at levels comparable to those typically used in agricultural scenarios (Dobermann and Cassman, 2005; Defra, 2010). A synthetic fertilizer (Chempak^®^ Formula No. 3) was applied at a “high” (HN) and a “low” (LN) N dose, which equated to 0.31 and 0.62 g^-1^ pot^-1^ respectively, which is approximately equivalent to 68 and 136 kg ha^-1^. The organic fertilizer was pelleted chicken manure (CM) (New Horizon Organic Poultry Manure Pellets), which was applied at the low N rate only (1.64 g^-1^ pot^-1^) since the quantity required to achieve the high N concentration would be excessive in relation to field application rates. Controls (CON) received the equivalent volume of water only. Chemical analysis of the fertilizers was carried out by NRM Laboratories Ltd. The synthetic fertilizer Chempak^®^ Formula No. 3 comprised 20.5% total N (w/w: 12.2% ureic N, 3.75% ammoniacal N, 4.56% nitric N), 20.6% water soluble P (as P_2_O_5_), and 21.2% K (as K_2_O); and the New Horizon Organic Poultry Manure Pellets contained 3.91% total N, 2.93% total P (as P_2_O_5_), and 2.47% total K (as K_2_O). Fertilizers were applied to each pot in an aqueous solution (50 ml pot^-1^), and the pots were kept in trays that were watered regularly. Each tray contained six pots from the same fertilizer treatment, to prevent fertilizer leachates reaching non-target pots. Bulk soil samples for DNA extraction were taken from the top 3 cm of the potted soil before and after the fertilizer additions.

### *Brassica oleracea* Cultivation, Aphid Infestation, and Soil Sampling

**FIGURE 1:**
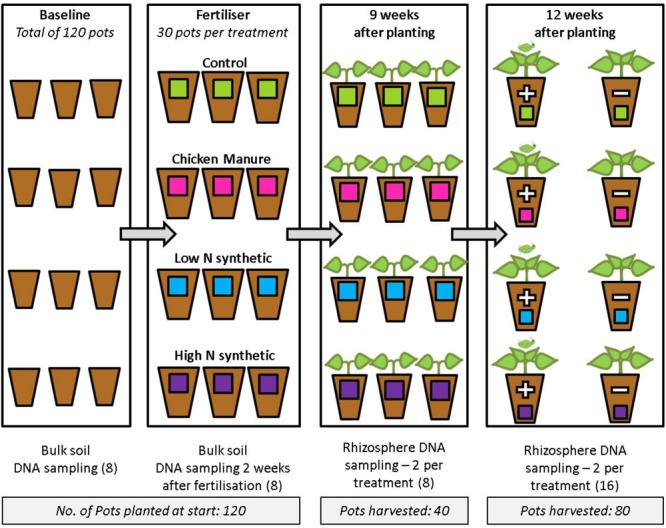
Experimental design showing stages at which DNA sampling was carried out. Each pot represents 10 replicates and each DNA sample was obtained from a pooled sample of soil taken from five pots. Numbers in brackets represent the total number of DNA samples taken at each sampling stage that were subsequently sequenced on the Illumina MiSeq. At the final (12 weeks) sampling stage, the “+” indicates plants infested with aphids, and “–” indicates non-infested plants.

The experiment was conducted in a controlled environment room with settings of 16:8 h light:dark, 20°C and 70% relative humidity. *B. oleracea* var. *capitata* L. cultivar Derby Day seeds [Moles Seeds (UK), Ltd] were sown after fertilizer application (5 seeds pot^-1^). Successfully germinated seedlings of similar size were then transplanted into the experimental pots of the corresponding fertilizer treatment, resulting in a total of 30 plants per treatment. This was done to allow for variances in germination success, and to ensure that any plant-specific accumulative effects on the soil microbiome were not confounded by differing numbers of germinated seeds in each pot. Soil adhering to the roots of the seedlings was included in the transplantation to minimize the disruption to seedling microbial recruitment. The plants were destructively harvested after either 9 or 12 weeks, with half of the plants in the latter harvest group being subjected to *M. persicae* herbivory (**Figure 1**). For aphid-infested plants, five apterous adult aphids were added to each plant using a paintbrush, and allowed to feed and reproduce for 14 days before harvesting at 12 weeks. The *M. persicae* colony was reared on Chinese cabbage *Brassica rapa* L. spp. *Pekinensis* (Lour) Cv. Wong Bok (Kings Seeds, Surrey, United Kingdom) in Perspex cages (70 cm × 69 cm × 45 cm) under controlled environment conditions (20 ± 3°C, 16:8 h light:dark). These harvest points were significant since the concentrations of total phenols and flavonoids in white cabbage heads have been reported to rapidly increase until reaching a peak at 12 weeks after planting (Šamec et al., 2011). Concomitantly, there is an increase in antioxidant capacity between 8 and 12 weeks, and therefore this growth stage may signify a more active metabolism of the plant. *M. persicae* mean relative growth rate (*r_m_*) has been shown to be significantly negatively correlated with total phenol content of bell pepper leaves (Mardani-Talaee et al., 2016), while enhanced antioxidant activity has been reported as a defense response of pea seedlings to attack by the pea aphid *Acyrthosiphon pisum* (Morkunas et al., 2016). Therefore, it was expected that differences in plant susceptibility to aphids between fertilizer treatments would be most pronounced during this growth stage.

At each sampling stage, two soil samples were collected from each treatment group. Each of these soil samples consisted of soil pooled from five pots (within a treatment group) in order to improve the accuracy of the bacterial community represented by each sample and to reduce the effects of the small sample size. Rhizosphere soil was classed as the soil still attached to the roots after they had been placed in a plastic bag and shaken vigorously in order to remove the adhering bulk soil (note that bulk soil was sampled during the pre-planting stages only). The roots were then placed in 25 ml distilled water and centrifuged (3000 × *g*, 15 min) twice. A subsample (0.25 g) of the resultant pellet was used for DNA isolation.

### Plant Yield and Foliar Nutrient Content

The fresh biomass is reported for all plants harvested at 9 (*n* = 10, except HN *n* = 7 owing to three plant deaths) and a subset of those harvested at 12 weeks (without aphids *n* = 3; with aphids *n* = 5). Three non-infested, 12-week-old plants from each treatment were analyzed for total foliar N and C content by NRM Laboratories, Ltd. This was accomplished via the Dumas method, which involved total combustion of the dried and ground (<0.5 mm) plant samples in an oxygen enriched atmosphere, the products of which were then passed through a thermal conductivity detector. The electronic signal produced by the detector signifies the amount of N and C present. These plants were also used for the fresh biomass measurements for 12-week-old, uninfested plants.

### DNA Extraction and 16S rRNA Gene Sequencing

DNA was extracted from 0.25 g sub-samples of pooled soil using the PowerSoil^®^ DNA Isolation kit (Mo Bio Laboratories) following the protocol supplied by the manufacturer. A total of 40 DNA samples were used for sequencing, comprising 16 samples of bulk soil (before and 2 weeks after fertilizer additions) and 24 samples of rhizosphere soil (from cabbages after 9 weeks growth and 12 weeks with or without aphids) (**Figure 1**). DNA samples were quality checked by 0.8% agarose gel electrophoresis and quantified fluorometrically using Qubit^®^. For the post-fertilizer bulk soil samples, a sample from both the CM and the high N treatments were not of sufficient quality for sequencing. Therefore, to utilize the full sequencing capacity available, an extra (third) DNA sample was taken from each of the control and low N treatments.

The library preparation and sequencing of the V4 hypervariable region of the prokaryotic (bacteria and archaea) 16S ribosomal RNA (rRNA) gene for all 40 samples was carried out by the Centre for Genomic Research (CGR) at the University of Liverpool using primers 515F (5′-GTGCCAGCMGCCGCGGTAA-3′) and 806R (5′-GGACTACHVGGGTWTCTAAT-3′). Further details are given in the Supplementary Material.

De-multiplexing and initial processing of the resultant sequencing library was performed by the University of Liverpool’s CGR using an in-house pipeline. This was done using CASAVA version 1.8.2 (Illumina) to de-multiplex samples, Cutadapt v.1.2.1 (Martin, 2011) to remove adapter sequences, and Sickle v. 1.200 (Joshi and Fass, 2011) to remove low-quality reads. The paired-end reads were assembled into a single read using FLASh v1.2.8 (Magoč and Salzberg, 2011). Downstream processing of the 16S rRNA libraries was performed in QIIME (Quantitative Insights Into Microbial Ecology) version 1.9.1 (Caporaso et al., 2010). Sequences were clustered into operational taxonomic units (OTUs) at 97% similarity and chimeric sequences were removed using USEARCH (Edgar, 2010). Open-reference OTU picking was performed using the command *pick_open_reference_otus.py* and the Greengenes version 13.8 database (McDonald et al., 2012) was used to classify representative OTUs. Sequences identified as singletons (only occurring once), or belonging to chloroplast or mitochondria were discarded. To account for between-sample differences in sequencing depth, data were normalized to 198,288 sequences per sample (the minimum number in a single sample).

All trimmed sequence reads generated in this study are available in the Sequence Read Archive (SRA) database of the National Centre for Biotechnology Information (NCBI) under accession number SRP134261.

### Statistical Analysis

Statistical analysis was performed in QIIME (MacQIIME v1.9.1) (Caporaso et al., 2010), STAMP v2.1.3 (Parks et al., 2014), and R v3.3.0 (R Development Core Team, 2008). First, a broader assessment of the diversity within (alpha) and between (beta) groups was conducted. Alpha (α) diversity was measured using the metrics Chao1 richness, Faith’s phylogenetic diversity (PD) (Faith, 1992), and observed OTUs (species). Beta (β) diversity was assessed using UniFrac distances (weighted and unweighted) and Bray–Curtis dissimilarities based on normalized (rarefied) OTU abundances. UniFrac (Lozupone and Knight, 2005) is a phylogenetically based distance metric, whereas Bray–Curtis dissimilarity distances are determined solely by the taxonomic composition (abundance) of the community regardless of phylogenetic relatedness. Thus, if the differences in community structure between groups are due to taxa that are (phylogenetically) closely related, they are more likely to be detected by Bray–Curtis rather than UniFrac distances. Weighted UniFrac distances account for both the presence and abundance of taxa, whereas unweighted UniFrac is determined solely by the presence or absence of OTUs.

The statistical analysis of β-diversity was structured around the four approaches recommended by Anderson and Willis (2003). These were (1) an unconstrained ordination, (2) a constrained ordination, (3) statistical testing of the main hypothesis, and (4) identification of the main taxa driving the main differences. The precise methods used in this study according to these four approaches were (1) detrended correspondence analysis (DCA), (2) canonical analysis of principal coordinates (CAP), (3) permutational multivariate analysis of variance (PERMANOVA), and (4) determination of key drivers (i.e., taxonomic groups) responsible for any observed differences by statistical tests (DESeq2). DCA (unconstrained) and CAP (constrained) ordinations were calculated in R using the *capscale* function in the *vegan* package. Statistical differences between the β-diversities of soil communities according to treatment and sample type were evaluated using the *vegan, phyloseq*, and *ggplot2* packages in R. The statistical significance of the ordinations was tested using PERMANOVA with 9,999 permutations via the *adonis* function of the *vegan* package. PERMDISP was also performed to test the homogeneity of the sample groups using the *betadisper* function in the vegan package in R. Finally, the DESeq2 package (Love et al., 2014) was used to detect pairwise differences in taxonomic abundances based on cabbage age (9 or 12 weeks), aphid presence (with or without aphids at 12 weeks), and fertilizer treatment (pairwise comparisons between control, CM, low N, high N of the same harvest age). Differences were deemed significant if they met two criteria: (i) a log2 fold change threshold of 1.2 and (ii) *p*-value cut-off of 0.01 (adjusted for false discovery rate using Benjamini–Hochberg correction), thus limiting it to taxa which differed by at least 20% with a 2% chance of false positive identification. Samples from different fertilizer groups were analyzed separately when testing for effects of plant age and aphid herbivory. As no significant effect of aphid herbivory was found, the 12-week samples for infested and uninfested plants within each treatment were combined in the DESeq2 analysis. See Supplementary Material for further details of the statistical methods used.

## Results

### Plant Yield and Nutrient Content

**FIGURE 2:**
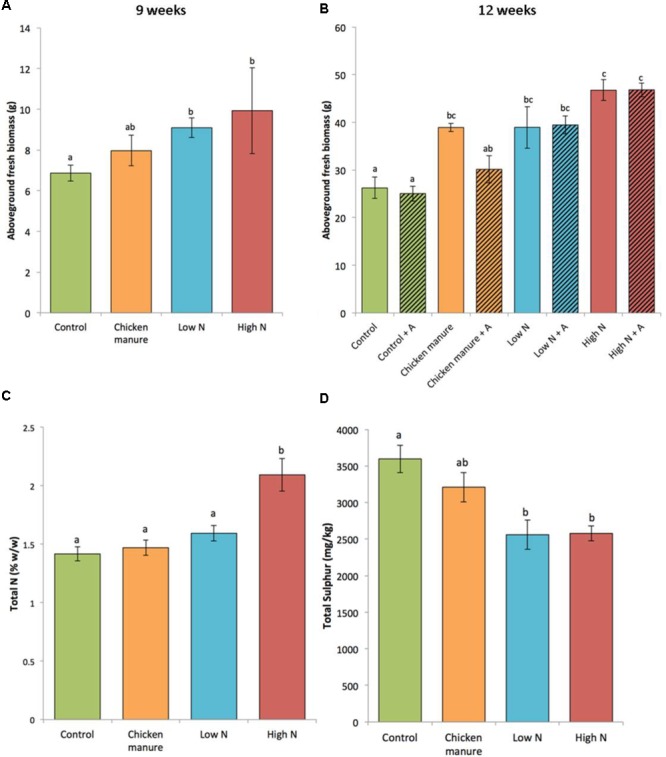
Comparison of the plant growth parameters under different fertilizer and aphid treatments (+A indicates plants infested with aphids) (mean ± standard error). **(A)** Aboveground fresh weight of 9-week-old plants (*n* = 10, except HN *n* = 7); **(B)** aboveground fresh weight of 12-week-old plants without (open bars, *n* = 3) and with aphids (hatched bars, *n* = 5); **(C)** total foliar nitrogen (N) of 12 week-old plants (*n* = 3); **(D)** total foliar sulfur (S) of 12-week-old plants (*n* = 3). Different letters above the bars indicate significant differences.

Fertilizer treatments resulted in significantly different fresh biomass of plants harvested at 9 weeks [one-way test (not assuming equal variances) *F*_3,33_ = 4.2261, *p* = 0. 0227] (**Figure 2A**). Synthetically fertilized 9-week-old plants (LN and HN) had a significantly greater aboveground biomass than controls (Dunn’s test Con-HN *p* = 0.0450 and Con-LN *p* = 0.0293). A two-way ANOVA also revealed a significant fertilizer effect on the fresh weight of 12-week-old cabbages (*F*_3,24_ = 31.274, *p* < 0.001), but no effect of aphid herbivory was detected (*F*_1,24_ = 1.986, *p* = 0.172) (**Figure 2B**). No significant interaction was detected between aphid herbivory and fertilizer treatment on plant biomass (two-way ANOVA *F*_3,24_ = 1.702, *p* = 0.193). *Post hoc* Tukey HSD tests showed that the control plants had a significantly lower fresh weight biomass than those treated with CM (*p* = 0.0098), Low N (*p* < 0.0001), and High N (*p* < 0.0001). High N plants had a greater biomass than CM (*p* < 0.0001) and Low N (*p* = 0.0147) plants. There was no significant difference in the fresh biomass of Low N and CM plants (*p* = 0.0761).

Fertilizer treatments had a significant effect on total foliar N in 12-week-old plants (**Figure 2C**) (one-way ANOVA: *F*_3,8_ = 12.1, adjusted *R*^2^ = 0.7517, *p* = 0.0024). As expected, foliar N content was significantly higher in plants from the High N treatment than all other treatments (Tukey’s HSD: *p* < 0.05), while the control plants had the lowest concentration (1.4167% w/w). In comparison to controls, the average foliar N content was increased by 47.8, 12.5, and 3.8% in HN, LN, and CM plants, respectively.

The sulfur (S) content of plants exhibited a reversal of this trend, with the LN and HN plants having significantly lower S content than control plants (one-way ANOVA: *F*_3,8_ = 8.018, *p* = 0.0085; Tukey’s HSD test: *p* < 0.05 for both HN and LN comparisons with control) (**Figure 2D**).

### Overview of the Bacterial Community

A total of 11,490,536 sequences corresponding to the V4 region of the prokaryotic (bacteria and archaea) 16S rRNA gene were obtained for all 40 samples, with a mean of 287,263 (±42,642) per sample. After clustering of these sequences, 82,460 archaeal and bacterial OTUs were identified. The number of sequence reads per sample was rarefied to 198,288 in order to normalize the data set and account for sampling bias. Overall, the 10 dominant phyla (mean relative abundance per sample) were *Proteobacteria* (27.2%), *Acidobacteria* (25.9%), *Planctomycetes* (9.5%), *Actinobacteria* (8.3%), *Bacteroidetes* (7.0%), *Chloroflexi* (6.0%), *Verrucomicrobia* (5.1%), *Gemmatimonadetes* (2.3%), *WS3* (2.1%), and *Nitrospirae* (1.4%) (Supplementary Material). Combined, these 10 phyla accounted for 94.7% of total bacterial abundance in all samples, and they are consistent with soil bacterial communities reported in other studies (Shang et al., 2016; Yang et al., 2017).

### Alpha-Diversity

There was no significant effect of fertilizer additions on α-diversity for any of the metrics employed (*p* > 0.05). However, the Low N and High N synthetic fertilizer treatments had the lowest values for each α-diversity measure for 9- and 12-week-old plants, respectively. There were no significant differences in the α-diversity when testing for the effects of cabbage age (**Table 1**) or aphid presence (**Table 2**).

**Table 1:** Alpha diversity metrics at cabbage growth stages [week 9 and 12 (no aphids) harvest] using a non-parametric two-sample *t*-test using Monte Carlo permutations [Mean (*± SD*)].

	9 Weeks		12 Weeks		*t*-Statistics	*p*-value
Chao1	24,194.51	±*1879.53*	22,859.90	±*1950.05*	1.3038	0.2220
Faith’s PD	811.63	±*67.71*	789.80	±*47.85*	0.6965	0.4890
Observed OTUs	15,869.56	±*1053.81*	15,238.43	±*954.38*	1.1745	0.2680
Simpson	0.9988	±0.0001	0.9987	±0.0002	–1.5581	0.972

*SD, standard deviation; PD, phylogenetic diversity; OUTs, operational taxonomic units.*

**Table 2:** Alpha diversity metrics for aphid-infested and aphid-free 12-week-old cabbages using a non-parametric two-sample *t*-test using Monte Carlo permutations [Mean (*±SD*)].

	With aphids		No aphids		*t*-Statistics	*p*-value
Chao1	22,007.20	±*1724.79*	22821.11	±*2034.33*	–0.8074	0.4460
Faith’s PD	770.08	±*40.33*	790.00	±*48.30*	–0.8376	0.4230
Observed OTUs	14,818.43	±*833.26*	15238.49	±*957.59*	–0.8755	0.4010
Simpson	0.9986	±0.0001	0.9987	±0.0002	0.7046	1

*SD, standard deviation; PD, phylogenetic diversity; OUTs, operational taxonomic units.*

### Beta-Diversity

The beta (β)-diversity analyses showed that cabbage age and fertilizer treatment were both found to have a significant effect on bacterial community composition of the rhizosphere according to all distance metrics used. There was also a significant interaction effect between fertilizer and cabbage age detected for all distance metrics, with the exception of unweighted UniFrac for which no significant interaction was detected (**Table 3**). There was no significant effect of aphid presence on β-diversity (**Table 4**).

**Table 3:** Results of permutational multivariate analysis of variance (PERMANOVA) analysis of dissimilarities for bacterial OTU community structure in relation to cabbage age, fertilizer treatment, and their interaction using UniFrac and Bray–Curtis distances.

Diversity metric	Statistic	Cabbage age (9 vs. 12 weeks no aphids)	Fertilizer	Cabbage Age^∗^Fertilizer Interaction
Unweighted	Df	1, 15	3, 15	3, 15
UniFrac	SS	0.1112	0.3446	0.2851
	MS	0.1112	0.1149	0.0950
	*F*-value	1.2374	1.2779	1.0574
	*R*^2^	0.0762	0.2360	0.1953
	*p*-value	**0.026^∗^**	**0.001^∗∗∗^**	0.17

Weighted	Df	1, 15	3, 15	3, 15
UniFrac	SS	0.0603	0.0687	0.0682
	MS	0.0603	0.0229	0.0227
	*F*-value	5.8009	2.2007	2.1841
	*R*^2^	0.2152	0.2449	0.2431
	*p-*Value	**0.001^∗∗∗^**	**0.016^∗^**	**0.011^∗^**

Bray–Curtis	Df	1, 15	3, 15	3, 15
	SS	0.08361	0.1602	0.1311
	MS	0.08361	0.0534	0.0437
	*F*-value	2.5576	1.6331	1.3365
	*R*^2^	0.13139	0.2517	0.2060
	*p*-value	**0.001^∗∗∗^**	**0.001^∗∗∗^**	**0.033^∗^**

*Df, degrees of freedom; SS, sum of squares; MS, mean sum of squares; *R*^2^, % variation explained; *F*-value, *F* value by permutation. Bold face indicates statistical significance (*p* < 0.05) and asterisks indicate the degree of the significance. *p*-values are based on 999 permutations (i.e., the lowest possible *p*-value is 0.001).*

**Table 4:** Results of permutational multivariate analysis of variance (PERMANOVA) analysis of dissimilarities for bacterial OTU community structure of 12 week cabbage rhizospheres in relation to herbivory (±aphids), fertilizer treatment and their interaction using UniFrac and Bray–Curtis distances.

Diversity metric	Statistic	Herbivory (±aphids)	Fertilizer	Herbivory^∗^Fertilizer
Unweighted	Df	1, 15	3, 15	3, 15
UniFrac	SS	0.0945	0.3232	0.2755
	MS	0.0945	0.1077	0.0918
	*F*-Value	1.0128	1.1541	0.9838
	*R*^2^	0.0657	0.2244	0.1913
	*p*-Value	0.335	**0.001**	0.678

Weighted	Df	1, 15	3, 15	3, 15
UniFrac	SS	0.0095	0.0804	0.0324
	MS	0.0095	0.0268	0.0108
	*F*-Value	0.7984	2.2632	0.9134
	*R*^2^	0.0436	0.3705	0.1495
	*p*-Value	0.609	**0.005^∗∗^**	0.587

Bray–Curtis	Df	1, 15	3, 15	3, 15
	SS	0.0339	0.1752	0.0986
	MS	0.0339	0.0584	0.0329
	*F*-Value	0.9365	1.6137	0.9088
	*R*^2^	0.0567	0.2933	0.1652
	*p*-Value	0.601	**0.001^∗∗∗^**	0.789

*Df, degrees of freedom; SS, sum of squares; MS, mean sum of squares; *F*-value, *F*-value by permutation; *R*^2^, % variation explained. Bold face indicates statistical significance (*p* < 0.05) and asterisks indicate degree of significance. *p*-values are based on 999 permutations (i.e., the lowest possible *p*-value is 0.001).*

A permutation test for homogeneity of multivariate dispersions (PERMDISP) (Anderson, 2006) was subsequently used to test for multivariate homogeneity of dispersions for each of the groups that yielded significant PERMANOVA results (Supplementary Material). The PERMDISP results indicated that the group dispersions of β-diversity calculated with the UniFrac (unweighted and weighted) and Bray–Curtis distances were not significantly different for any of the explanatory variables (cabbage age, herbivory, fertilizer treatment), thus any significant differences detected by the PERMANOVA test can be attributed to differences in their centroid.

Detrended correspondence analysis performed using UniFrac distances indicated divergence in the bacterial community composition of samples grouped by sample type (Supplementary Material). The DCA analysis also showed apparent grouping of the samples according to soil type, with clear separation of the rhizosphere and bulk soil samples (although note that these were collected at different time points). The main areas of overlap occurred between the aphid and no aphid rhizosphere samples (both taken from cabbages harvested simultaneously at 12 weeks), which appear to diverge slightly from the 9-week cabbage rhizosphere samples. This suggests, therefore, a temporal shift occurred in the rhizosphere community composition during plant development.

**FIGURE 3:**
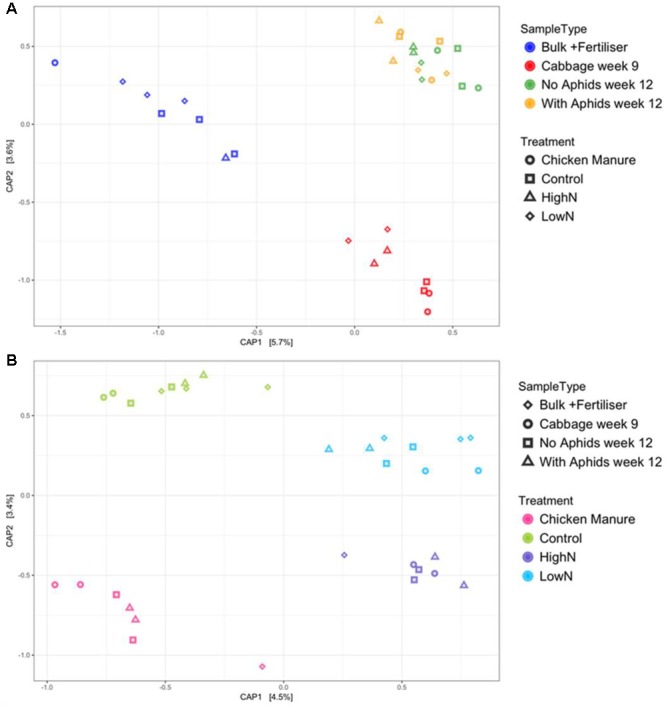
Canonical analysis of principal coordinates (CAP) plots created using unweighted UniFrac measures grouped by **(A)** sample type and **(B)** fertilizer treatment. PERMANOVA with 9999 permutations: **(A)** Sample type: *p* = 0.0001, *F*_3,28_ = 1.3008, SS = 0.3937; **(B)** Fertilizer treatment: *p* = 0.0088, *F*_3,28_ = 1.1458, SS = 0.35191.

The DCA plot indicated that linear ordination methods should be used since the first DCA axis was less than 3 units in length. The chosen method of constrained (linear) ordination was canonical PCoA (CAP) using unweighted UniFrac distances (**Figure 3**). When using sample type as the predicting factor, all the rhizosphere samples clustered together on the first axis of the CAP plot, away from the bulk soil samples (**Figure 3A**). The rhizosphere samples formed two distinct clusters on the CAP2 axis according to plant age, with the 12-week samples (both with and without aphids) grouping together away from the 9-week-old rhizosphere samples. When performing the analysis with fertilizer treatment as the grouping factor, the CAP plot showed the synthetically fertilized soils (LN and HN) diverging away from the control and organically fertilized (CM) soils on the first axis (**Figure 3B**). The organically fertilized (CM) samples diverged away from the untreated control samples on the second axis only. In both CAP plots, it is interesting to note that samples in the LN and CM treatments (applied at the same total N dose) do not overlap.

### Fertilizer-Associated Bacteria

All three fertilizer treatments were associated with increased abundance of members of the phyla *TM7-1, Bacteroidetes, Verrucomicrobia, Proteobacteria*, and genera *Flavobacterium* and *Lysobacter*.

### Chicken Manure

**FIGURE 4:**
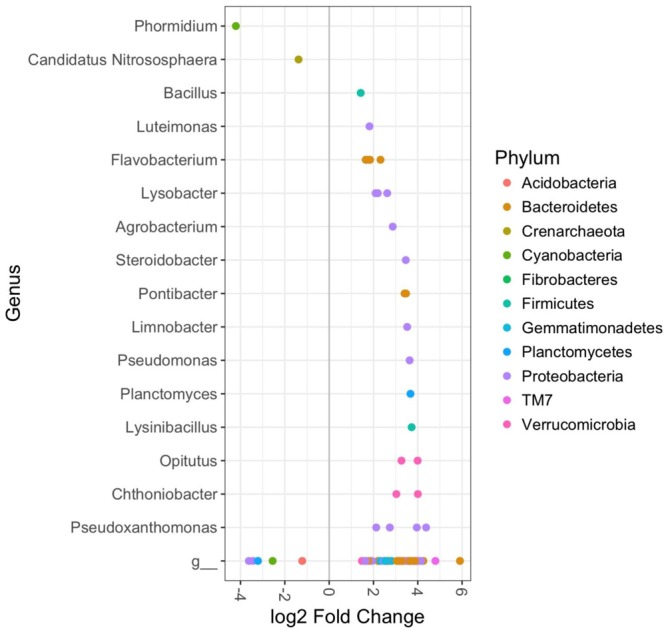
DESeq2 analysis results indicating the fold-change of bacterial genera in rhizosphere soil bacterial communities of cabbages in the Chicken Manure fertilizer treatment relative to control cabbages at 12 weeks (*p* < 0.01). Includes both aphid infested and uninfested plants as there were no significant effects of the aphids. “g” Represents taxa unclassified at the genus level.

The bacterial composition of rhizosphere soils in the CM treatment group were significantly different from controls in plants harvested at 12 weeks, but not 9 weeks (DESeq2 Benjamini–Hochberg corrected *p*-value < 0.001). This was largely due to a number of OTUs, which were significantly enriched in the organically fertilized soils. These included several members of the phyla *TM7, Proteobacteria*, and *Verrucomicrobia*; and the genera *Pseudoxanthomonas, Chthoniobacter, Opitutus, Planctomyces, Lysobacter, Flavobacterium*, and *Luteimonas* (DESeq2 Benjamini–Hochberg corrected *p*-value < 0.001, **Figure 4**). OTUs that were significantly less abundant in the organically fertilized soils at 12 weeks (relative to controls) included the genus *Candidatus Nitrososphaera*.

### Synthetic Fertilizer

**FIGURE 5:**
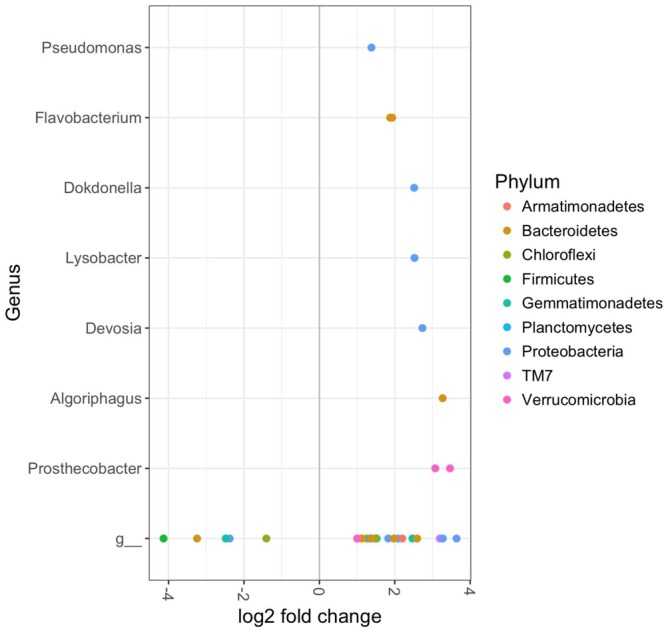
DESeq2 analysis results indicating the fold-change of bacterial genera in rhizosphere soil bacterial communities of cabbages receiving synthetic fertilizer treatments (Low N and High N) relative to control cabbages at 12 weeks (*p* < 0.01). Includes both aphid infested and uninfested plants as there were no significant effects of the aphids. “g” Represents taxa unclassified at the genus level.

OTUs enriched in the rhizospheres of synthetically fertilized plants (LN and HN) included the genera *Prosthecobacter, Algoriphaus, Devosia, Lysobacter, Dokdonella, Flavobacterium*, and *Pseudomonas* (**Figure 5**). Rhizosphere communities of 9-week-old LN plants exhibited increased abundances of OTUs assigned to the genera *Flavobacterium, Adhaeribacter, Arenimonas, Janthinobacterium, Sphingopyxis, Aequorivita, Fluviicola, Kaistobacter, Thermomonas*, and *Virgibacillus* (Supplementary Material). The LN week 9 soils had reduced abundance of several genera relative to control soils, including *Crocinitomix* and *Plesiocystis* (DESeq2 Benjamini–Hochberg corrected *p*-value < 0.001, Supplementary Material). There were also a several taxa with significantly different abundances in low N soils relative to CM soils at both 9 and 12 weeks (Supplementary Material), which indicates a significant effect of fertilizer type given these were applied at the same N dosage.

The HN-treatment affected rhizosphere soil communities at both 9 and 12 weeks. Relative to controls, the soils of 9-week-old HN cabbages were enriched in OTUs belonging to the genera *Sphingopyxis, Flavobacterium, Lysobacter, Algoriphagus, Arenimonas*, and *Kaistobacter* (DESeq2 Benjamini–Hochberg corrected *p*-value < 0.001, Supplementary Material). Several of these OTUs were assigned to the phylum *TM7* and the families *Flavobacteriaceae, Sphingomonadaceae*, and *Xanthomonadaceae*. At 12 weeks, the HN treated soils were enriched in several OTUs of unassigned species belonging to the genera *Sphingopyxis, Algoriphagus, Prosthecobacter, Chthoniobacter*, many *Flavobacterium, Pedobater, Methylotenera, Devosia, Microbacterium, Dokdonella, Lysobacter, Thermomonas, Planctomyces, Arenimonas*, many *Pseudomonas, Luteolibacter*, and *Luteimonas* (Supplementary Material). Also elevated in the HN soils of 12 week-old cabbages were OTUs belonging to the phyla *Gemmatimonadetes*; *Planctomycetes*, and *TM7*; orders *Myxococcales, Sphingobacteriales, Sphingomonadaceae*; and the families *Chitinophagaceae, Verrucomicrobiaceae*, and *Xanthomonadaceae* (DESeq2 corrected *p* < 0.05).

OTUs with significantly lower abundances in HN soils at 9 weeks included the genera *Plesiocystis* and *Phormidium*, while 12-week-old HN rhizosphere soils had comparatively less *Chrondromyces, Bdellovibrio*, and *Fibriimonas* (Supplementary Material). It was also noted that the rhizospheres of both groups of synthetically fertilized plants harvested in week 9 had considerably lower mean abundances of *Nitrospira* relative to control and organically fertilized soils (Supplementary Material). This genus contains species of nitrifying bacteria, which are important contributors toward emissions of the greenhouse gas nitrous oxide (N_2_O) from soils.

### Plant Growth Stage-Associated Rhizosphere Bacteria

**FIGURE 6:**
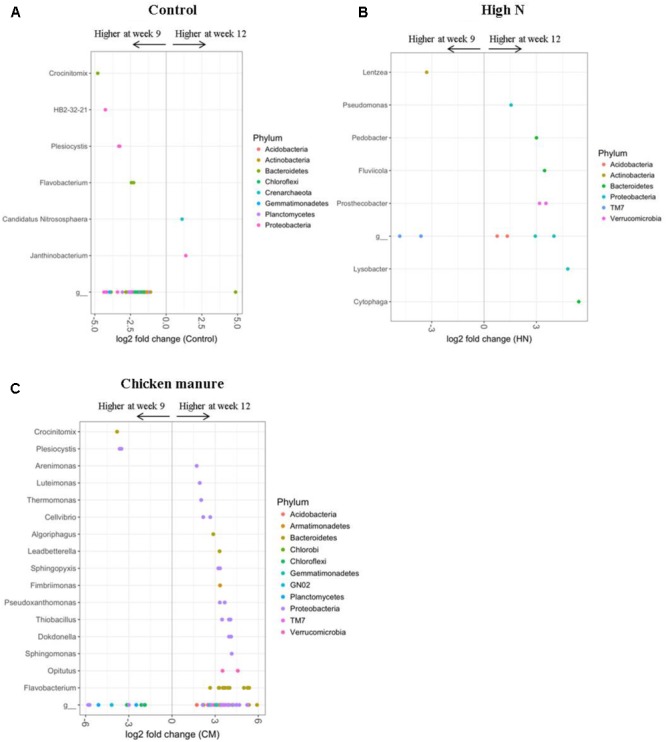
DESeq2 analysis results indicating the fold-change of bacterial genera in rhizosphere communities of 12-week-old plants relative to 9-week-old plants within the **(A)** control, **(B)** HN, and **(C)** CM treatments (LN treatment only had one significantly different classified OUT at the genus level) (*p* < 0.001). “g” Represents OTUs that were not classified at the genus level.

The soil bacterial communities of 9- and 12-week-old plants differed significantly in all fertilizer treatments, with the greatest age-related effects being detected in control, CM, and High N plants. In comparison to older (12 weeks) plants, the 9-week-old CON plants were enriched in many OTUs belonging to the genera *Crocinitomix, HB2-32-21, Plesiocystis*, and *Flavobacterium* (DESeq2 Benjamini–Hochberg corrected *p*-value < 0.001, **Figure 6A**). Older control plants had a higher abundance of OTUs from the phylum *Bacteroidetes*; genera *Candidatus Nitrososphaera* and *Janthinobacterium* (DESeq2 Benjamini–Hochberg corrected *p*-value < 0.001, **Figure 6A**).

In comparison to older plants, HN plants at 9 weeks were more abundant in only a few OTUs belonging to the phylum *TM7* and the genus *Lentzea* (DESeq2 Benjamini–Hochberg corrected *p*-value < 0.001, **Figure 6B**). OTUs which became more dominant in older HN plants included members of the phyla *Acidobacteria, Bacteroidetes, Proteobacteria*, and *Verrucomicrobia*; including the genera *Cytophaga, Lysobacter, Prosthecobacter, Fluviicola, Pedobacter*, and *Pseudomonas* (DESeq2 Benjamini–Hochberg corrected *p* < 0.001, **Figure 6B**).

**FIGURE 7:**
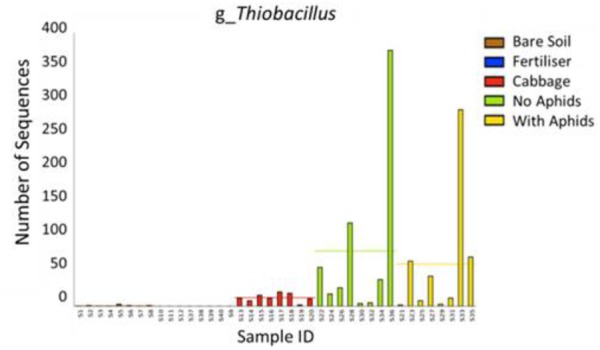
Comparison of the abundance of *Thiobacillus* sequences detected in each sample. They were almost exclusively found in rhizosphere soil samples [cabbage rhizosphere at 9 weeks (red), 12 weeks without aphids (green), and 12 weeks with aphids (yellow)].

In the CM treatment, 12-week-old plants were enriched in many OTUs assigned to the phyla *Bacteroidetes* and *Proteobacteria* in comparison to younger (9 weeks) CM plants. At the genus level, these older plants were enriched in *Flavobacterium, Opitutus, Sphingomonas, Dokdonella, Thiobacillus, Pseudoxanthomonas, Fimbriimonas, Sphingopyxis, Leadbetterella, Algoriphagus, Cellvibrio, Thermomonas, Luteimonas*, and *Arenimonas* (DESeq2 Benjamini–Hochberg corrected *p*-value < 0.001, **Figure 6C**). OTUs which appeared to decline in abundance with plant age in the CM treatment included the genera *Crocinitomix* and *Plesiocystis* (DESeq2 Benjamini–Hochberg corrected *p*-value < 0.001, **Figure 6C**). The sulfur-oxidizing bacteria *Thiobacillus* was almost exclusively found in rhizosphere samples, and absent from the majority of bulk soil samples (**Figure 7**). In particular, it appeared to be positively associated with the growth of organically fertilized *B. oleracea*, as it formed a larger proportion of the bacterial community in 12-week-old CM cabbages relative to those harvested at 9 weeks (**Figure 6C**). There were no significant differences in relative abundances of bacteria detected between cabbages with or without aphids.

## Discussion

This study shows, for the first time, that the rhizosphere community of *B. oleracea* var. *capitata* cv. Derby Day is significantly altered by plant age and fertilizer regime. Fertilizer type (organic or synthetic) appeared to produce a stronger bacterial response than the quantity of fertilizer added (nitrogen dose), while aphid herbivory appeared to have no influence on the rhizosphere community.

### Fertilizer Effects on Plant Performance

This strength of effect of fertilizer type observed in the bacterial community was not reflected in the plant response. Plant yield (fresh biomass) did not differ significantly between CM and LN plants at either harvest time and, although slightly higher, total foliar N levels were not significantly different between the Low N synthetically fertilized plants and the organically fertilized plants (Tukey’s HSD *p* > 0.05). This suggests that the type, or *quality*, of fertilizer applied is less influential on plant performance than the dose, or *quantity*, since the two fertilizers (LN and CM) were applied at the same total N rate. This lack of significance contradicts previous studies which indicated that plants treated with synthetic fertilizers have significantly higher foliar N levels than organically fertilized plants (Costello and Altieri, 1995; Morales et al., 2001; Staley et al., 2011). However, as only three plants per treatment group were used for the chemical analysis in this study, this absence of a significant effect may be a consequence of low sample numbers.

The inverse relationship found between foliar N and S content of 12-week-old plants concurs with previous findings that plant S concentrations decline with increasing N additions (and vice versa), possibly due to a growth-dilution effect (Janzen and Bettany, 1984; McGrath and Zhao, 1996; Schonhof et al., 2007). This apparent inhibition of S uptake in synthetically fertilized plants may have a significant bearing on the plant’s defenses against aphid herbivory, given that sulfur is a major component of glucosinolate compounds. This theory is supported by the findings of a field experiment by Staley et al. (2010), in that the glucosinolate concentrations of organically fertilized plants were up to three times higher than in plants receiving mineral fertilizers.

### Fertilizer-Associated Bacteria

Although no statistically significant differences were detected in α-diversity, there were clear fertilizer effects on β-diversity shown in the CAP ordination, as synthetically fertilized soils diverged away from the control and organically fertilized soil samples. The PERMANOVA results supported these findings, as the fertilizer treatments and cabbage age were both found to have a significant effect on β-diversity both separately and as an interaction. Both synthetic and organic fertilizer treatments resulted in elevated abundance of several OTUs assigned to the phyla *Bacteroidetes* (including the genus *Flavobacterium*) and *Proteobacteria* (including the genus *Lysobacter*). *Lysobacter* belong to the order *Xanthomonadales*, which has been reported to respond positively to synthetic and organic fertilizers in other studies, with the impact penetrating into the subsoil (>0.2 m) (Campbell et al., 2010; Li et al., 2014a). Several flavobacteria are known to have denitrifying properties (Pichinoty et al., 1976; Horn et al., 2005), and this positive association with fertilizer treatments concurs with reports that denitrification rates increase following fertilizer additions (Bremner, 1997; Mulvaney et al., 1997), raising concerns that these farming regimes contribute to greenhouse gas (NO and N_2_O) emissions.

Plant N-uptake has a strong impact on the composition of microbial communities, with greater N-uptake resulting in lower bacterial diversity (Bell et al., 2015). It was expected, therefore, that the form of N (organic or inorganic) would influence the shaping of rhizosphere communities owing to differences in their availability for assimilation by plant roots. This was supported by our results, which showed a clear distinction between the rhizosphere communities of plants grown in organically and synthetically fertilized, as discussed below.

#### Organic Fertilizer

The organic fertilizer treatment was found to have a significant effect on bacterial abundances in the rhizosphere soils of 12-week-old cabbages only. The lack of an effect at 9 weeks may be due to the slow nutrient release rate common to organic fertilizers causing a delayed response in the bacterial community. Relative to controls, OTUs which proliferated in soils amended with poultry pellets included members of the genera *Chthoniobacter, Opitutus, Pseudoxanthomonas*, and *Planctomyces*. Species of *Pseudoxanthomonas* are capable of organic matter degradation (Kim et al., 2008), and have previously been isolated from cotton waste composts (Weon et al., 2006), fermented cow manure (Giannattasio et al., 2013), and soil fertilized with pig manure (Ding et al., 2014). This may, therefore, represent a common bacterial community response to a variety of animal-derived and plant-based organic fertilizers.

Soils treated with poultry manure pellets exhibited a significantly lower abundance of bacteria in the genus *Candidatus Nitrososphaera*. *Candidatus Nitrososphaera* is an ammonia-oxidizing archaea which has previously been found to be significantly higher in agricultural soils relative to those from non-agricultural sites (Zhalnina et al., 2013). The contradictory finding in our study may be attributed to the negative correlation between *Candidatus Nitrososphaera* with ammonium (the predominant form of N in poultry manure) and soil organic matter (SOM), as reported by Zhalnina et al. (2013). A reduction in ammonia-oxidizing microorganisms may be favorable in environmental terms since they contribute toward emissions of the highly potent greenhouse gas nitrous oxide (N_2_O) from agricultural soils (Stieglmeier et al., 2014).

#### Synthetic Fertilizer

The rhizomicrobiomes of synthetically fertilized 12-week-old plants were enriched several members of the *Proteobacteria, Verrucomicrobia*, and *Bacteroidetes*, such as the genera *Prosthecobacter, Algoriphagus, Devosia, Lysobacter, Dokdonella, Flavobacterium*, and *Pseudomonas*. *Flavobacterium* bacteria perform heterotrophic denitrification (Wang et al., 2014) and their abundance has previously been shown to increase in soils under conventional (chemical), but not organic, management (Lavecchia et al., 2015).

In comparison to controls, the rhizospheres of 9-week-old synthetically fertilized plants had a significantly lower abundance of the nitrifying bacteria of the genus *Nitrospira*. This agrees with previous reports of chemically fertilized soils exhibiting significantly smaller *Nitrospira* populations in comparison to control and organically managed soils (Simonin et al., 2015; Ding et al., 2016; Eo and Park, 2016). Both LN and HN synthetically fertilized soils also had a diminished prevalence of bacteria in the genera *Phormidium* in comparison to controls. *Phormidium* are cyanobacteria commonly found in biological soil crusts, including those from very cold (e.g., Antarctic) and arid (e.g., Tibetan deserts) environments (Williams et al., 2016; Lacap-Bugler et al., 2017). These cyanobacteria have nitrogen-fixing properties and have been tested in a mix comprising other N-fixing cyanobacteria for their potential as a biofertilizer. It has been demonstrated that incorporating a *Phormidium*-containing inoculum into NPK fertilizer significantly increases yield, micronutrient content and leaf chlorophyll levels in wheat (Renuka et al., 2017). It is likely, therefore, that these bacteria thrive in nutrient-starved environments; with populations declining when N-availability is increased, as observed in our study.

### Rhizosphere Community Responses to Plant Growth

There was a clear effect of plant age on the composition of rhizomicrobiome on β-diversity, however there was no effect on α-diversity according to the metrics tested. The visualization of β-diversity via a constrained ordination method (CAP) showed a clear divergence between rhizosphere samples based on plant age. This concurs with other studies, such as that by Li et al. (2014a), which reported a distinct shift in rhizosphere microbial community composition over the course of plant development. DESeq analysis revealed that in comparison to older plants, younger (9-week-old) control, and CM plants were both richer in the genera *Crocinitomix* and *Plesiocystis*. As plants grow, the availability of nutrients in the soil is depleted which can lead to more oligotrophic-dominated soil communities. Older plants were associated with increases in the abundance of OTUs from the phyla *Bacteroidetes, Proteobacteria*, and *Verrucomicrobia*. Chaparro et al. (2014) found that the rhizospheres of *Arabidopsis* plants at the bolting/flowering stages exhibited increases in *Bacteroidetes* and *Cyanobacteria* in comparison to the seedling/vegetative growth stages. Li et al. (2014b) found a relationship between the rhizosphere community composition and the growth stage of maize plants, with *Chitinophaga* (phylum *Bacteroidetes*) being one of the more dominant bacteria during the later growth stage. Similarly, de Campos et al. (2013) found that *Xanthomonadaceae* (*Proteobacteria*) and *Flavobacteriaceae* (*Bacteroidetes*) dominated the bacterial community of canola (*Brassica napus* L. var *oleifera*) rhizospheres at the flowering stage. Our results show that several OTUs assigned to the genus *Flavobacterium* increased in abundance in the rhizospheres of CM plants over time. In nature *Flavobacterium* are known to mineralize organic substrates (e.g., carbohydrates, amino acids, and proteins) and degrade organic matter and other organisms (bacteria, fungi, and insects) using a variety of enzymes (Bernadet and Bowman, 2006; Kolton et al., 2016).

An interesting finding was that several OTUs assigned to the sulfur-oxidizing genus *Thiobacillus* (family *Hydrogenophilaceae*) were almost exclusively found in rhizosphere samples, particularly in the 12-week-old CM cabbages (**Figure 7**). *Thiobacillus* species are sulfur-oxidizing bacteria that grow in a wide range of conditions (optimum pH < 2–8 and temperature 20–50°C), deriving energy via the oxidation of one or more sulfur compounds including sulfides, thiosulfate, and thiocyanate (Kelly and Wood, 2000). *Thiobacillus thioparus* bacteria possess an enzyme that can breakdown thiocyanate – a common compound found in glucosinolates (Katayama et al., 1998). Furthermore, their growth has been shown to increase in response to dimethylsulfide (DMS), which is a by-product of the decomposition of *Brassica* biomass (Bending and Lincoln, 1999; Eyice and Schäfer, 2016). These observations may indicate that root-derived glucosinolates and decomposition derivatives play an important role in shaping the soil bacterial community of *Brassica* rhizospheres. *Thiobacillus* are also known for their ability to solubilize phosphorus, a valuable attribute given the importance of this nutrient in plant growth (Shen et al., 2011) and the continuing depletion of rock-organic phosphate resources, which are the main origin of P-fertilizers (Hunter et al., 2014). Indeed, the inoculation of soil with *Thiobacillus* has been shown to increase phosphorus availability (Boulif et al., 2016; Jazaeri et al., 2016), thus demonstrating its potential as a biofertilizer. Other studies have reported *Thiobacillus* species to be enriched in rhizosphere soils, including those of maize plants (Yang et al., 2017). It is possible that there is an affiliation between the rhizospheres of *Brassica* plants and P-solubilizing bacteria as another plant growth promoting species of phosphorus-solubilizing bacteria, *Bacillus cereus*, has previously been isolated from the rhizosphere of Chinese cabbage (*Brassica campestris* spp. *chinensis*) (Wang et al., 2017).

These results indicate that the rhizosphere community changes significantly during the development of *B. oleracea*. Plant growth stage has been shown to have a significant effect on the soil microbial community in a number of other plants, such as potato (*Solanum tuberosum*) (Pfeiffer et al., 2017) and maize (*Zea mays* L.) (Cavaglieri et al., 2009). Rhizodeposition of carbon-rich compounds (e.g., sloughed-off root border cells, mucilage, organic acids), a significant energy source for microbial growth, declines significantly with plant age (Nguyen, 2003; Chaparro et al., 2013). Although we found no significant effect of plant growth on α-diversity, other studies have reported that bacterial α-diversity (richness) of the rhizosphere microbiome declines with plant age (Liljeroth and Bååth, 1988; Chaparro et al., 2014; Shi et al., 2015), gradually converging with the bulk soil as the plant approaches senescence (Micallef et al., 2009). This temporal shift is thought to correspond to changes in the plant root exudate profile, with root exudates of *Arabidopsis* plants exhibiting decreasing levels of sugars and increasing amino acid and phenolic compounds over the course of plant development (Chaparro et al., 2014). Since sugars represent a major resource for microbial growth (Philippot et al., 2013) and phenolics often have antimicrobial properties (Barber et al., 2000), it may be expected that bacterial diversity is highest during the early stages of plant development. However, other studies report that root communities are robust and unaffected by the different phases of plant development (Dombrowski et al., 2017).

### Rhizosphere Community Response to Aphid Infestations

Although there was no effect of aphid herbivory on the rhizomicrobiome detected in this study, there have been other reports supporting such an interaction. Some studies have indicated that aphid herbivory reduces rhizosphere bacterial abundance, potentially due to negative effects of aphids on rhizodeposition rates and allocation of photoassimilates to roots (Vestergård et al., 2004). However, these effects on bacterial abundance can reverse at later stages of plant growth, as demonstrated by Vestergård et al. (2004) and Lee et al. (2012). Aphid infestations have been shown to correlate with increased abundance of the beneficial PGPR strain *Bacillus subtilis* GB03, and a reduction in the prevalence of the pathogenic *Ralstonia solanacearum* SL1931 (Lee et al., 2012). This was hypothesized to be caused by the above-ground herbivores (aphids) enhancing root exudation, thereby stimulating the recruitment of beneficial PGPR (Yang et al., 2011; Lee et al., 2012). Kim et al. (2016) also reported a change in root exudates of pepper plants following infestation with *M. persicae*, which was found to promote the growth of *Paenibacillus* species. The authors suggested that the plant actively recruits *Paenibaccilus* species in response to the aphid attack. Furthermore, a study by Kong et al. (2016) showed that, relative to control plants, whitefly infestations significantly altered the pepper rhizosphere after just 1 week. Alpha diversity was significantly lower in the rhizospheres of whitefly infested plants, which had significantly lower abundances of *Caulobacteraceae, Cytophagaceae, Oxalobacteraceae, Xanthomonadaceae*, and *Paenibacillaceae* relative to control plants. However, the pepper plants used in this study were just 2 weeks old when they were infested with whitefly and thus it may be that the impact of herbivory on root exudation/rhizosphere communities is dependent on plant age. Alternatively, it is possible that whitefly infestations have a stronger rhizosphere effect than aphids.

This study could have benefited from the monitoring of the bulk soil community throughout the experiment (i.e., sampled in parallel with rhizosphere sampling time points), as the bulk soil microbial composition can vary significantly over short timescales (Li et al., 2014b). However, some studies have shown that the microbial community varies less over time in comparison to other factors, such as spatial comparisons (Armstrong et al., 2016). Similarly, Shade et al. (2013) reported that soil microbial communities show little change over short/moderate time periods (<6 months). Nevertheless, this would merit further investigation to validate whether this is the case in our system. Further research is also required to determine whether the changes in the soil bacterial community observed in our study affects the functionality of the soil, which could be achieved using methods such as meta-transcriptomics.

## Conclusion

Our results show that the fertilizer treatments and Derby Day cabbage growth have a considerable impact on the soil bacterial community. High-throughput sequencing of the16S rRNA gene revealed that the rhizosphere community of this plant was significantly altered by fertilizer additions and harvest time. The finding that certain OTUs, such as the sulfur-oxidizing genus *Thiobacillus*, were almost exclusively found in the rhizosphere could represent a potential target for the biological fortification of human-beneficial plant attributes, such as glucosinolates concentrations.

## Author Contributions

FO designed and performed the experiments, analyzed the data, and wrote the manuscript. JW and GP contributed to the design of the experiments and writing of the manuscript. MD advised on data analysis and contributed to the writing of the manuscript.

## Conflict of Interest Statement

The authors declare that the research was conducted in the absence of any commercial or financial relationships that could be construed as a potential conflict of interest.
