# Supplementary material for: Rhizosphere Bacterial Communities Differ According to Fertilizer Regimes and Cabbage (Brassica oleracea var. capitata L.) Harvest Time, but Not Aphid Herbivory
Source: Front Microbiol. 2018 Jul 23;9:1620. doi: 10.3389/fmicb.2018.01620 (PMC6064718; doi:10.3389/fmicb.2018.01620)
Supplement: Supplementary file 1 [file Table_1.DOCX]

## Supplementary Table

Supplementary Table 1 Permutation test for homogeneity of multivariate dispersions (PERMDISP) of bacterial communities grouped by cabbage age, aphid herbivory and fertiliser treatment (999 permutations).

| **Grouping** | **Distance measure** | **D.F.** | **F-value** | ***p*-value** |
| --- | --- | --- | --- | --- |
| Fertiliser (+/- aphids 12 weeks) | Unweighted UniFrac | 3, 12 | 0.182 | 0.904 |
| Fertiliser (+/- aphids 12 weeks) | Weighted UniFrac | 3, 12 | 0.642 | 0.617 |
| Fertiliser (+/- aphids 12 weeks) | Bray Curtis | 3,12 | 0.418 | 0.743 |
| Herbivory (+/- aphids 12 weeks) | Unweighted UniFrac | 1, 14 | 2.003 | 0.154 |
| Herbivory (+/- aphids 12 weeks) | Weighted UniFrac | 1, 14 | 0.126 | 0.739 |
| Herbivory (+/- aphids 12 weeks) | Bray Curtis | 1,14 | 0.028 | 0.869 |
| Fertiliser (9 & 12 weeks no aphids) | Unweighted UniFrac | 3, 12 | 1.020 | 0.404 |
| Fertiliser (9 & 12 weeks no aphids) | Weighted UniFrac | 3, 12 | 2.393 | 0.094 |
| Fertiliser (9 & 12 weeks no aphids) | Bray Curtis | 3,12 | 1.469 | 0.272 |
| Cabbage age (9 & 12 weeks no aphids) | Unweighted UniFrac | 1, 14 | 0.543 | 0.5 |
| Cabbage age (9 & 12 weeks no aphids) | Weighted UniFrac | 1, 14 | 0.001 | 0.983 |
| Cabbage age (9 & 12 weeks no aphids) | Bray Curtis | 1,14 | 0.031 | 0.862 |
