# Supplementary material for: Rhizosphere Bacterial Communities Differ According to Fertilizer Regimes and Cabbage (Brassica oleracea var. capitata L.) Harvest Time, but Not Aphid Herbivory
Source: Front Microbiol. 2018 Jul 23;9:1620. doi: 10.3389/fmicb.2018.01620 (PMC6064718; doi:10.3389/fmicb.2018.01620)
Supplement: Supplementary file 2 [file Presentation_1.pptx]

## Slide 1
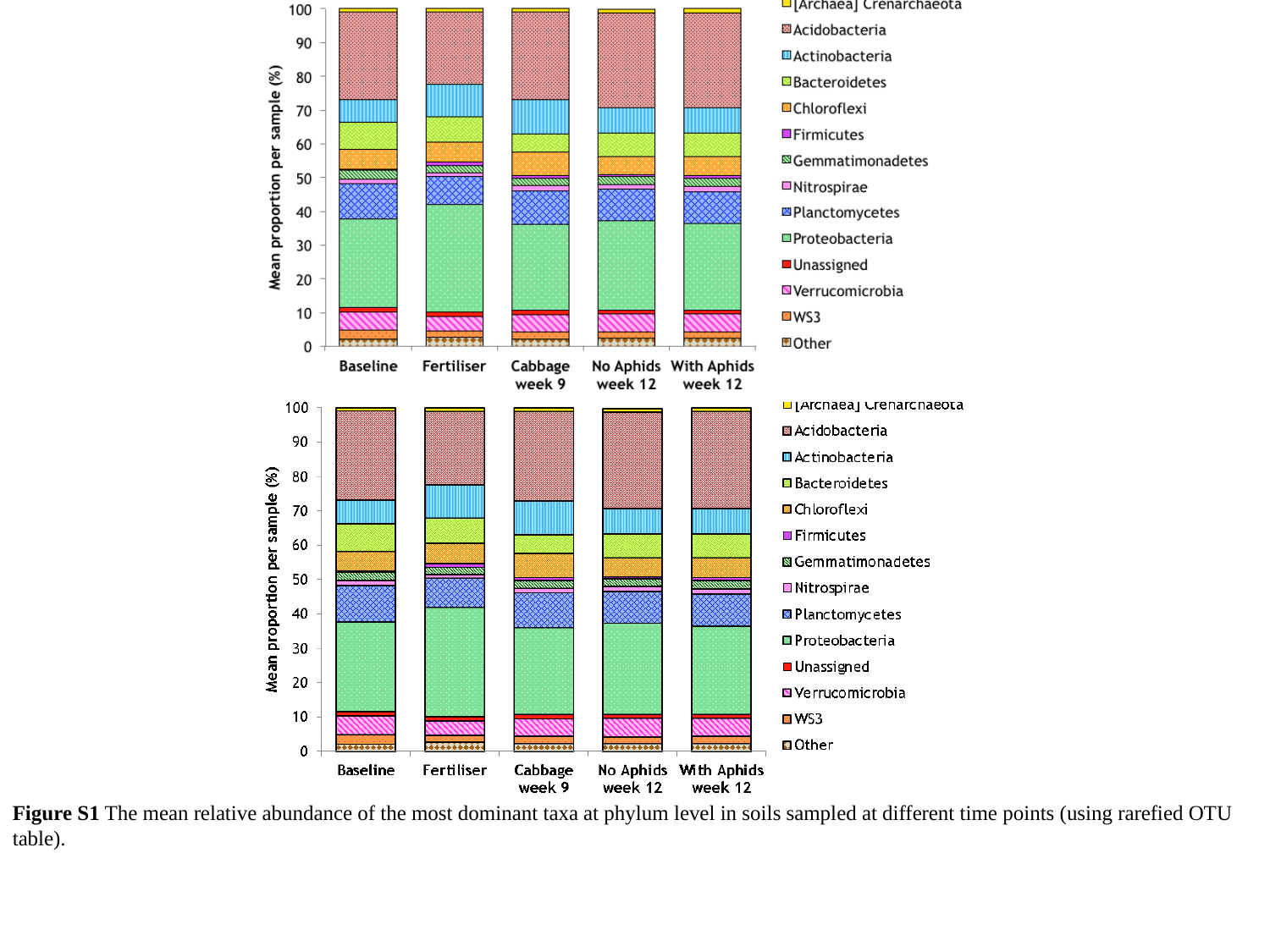

Figure S1 The mean relative abundance of the most dominant taxa at phylum level in soils sampled at different time points (using rarefied OTU table).

## Slide 2
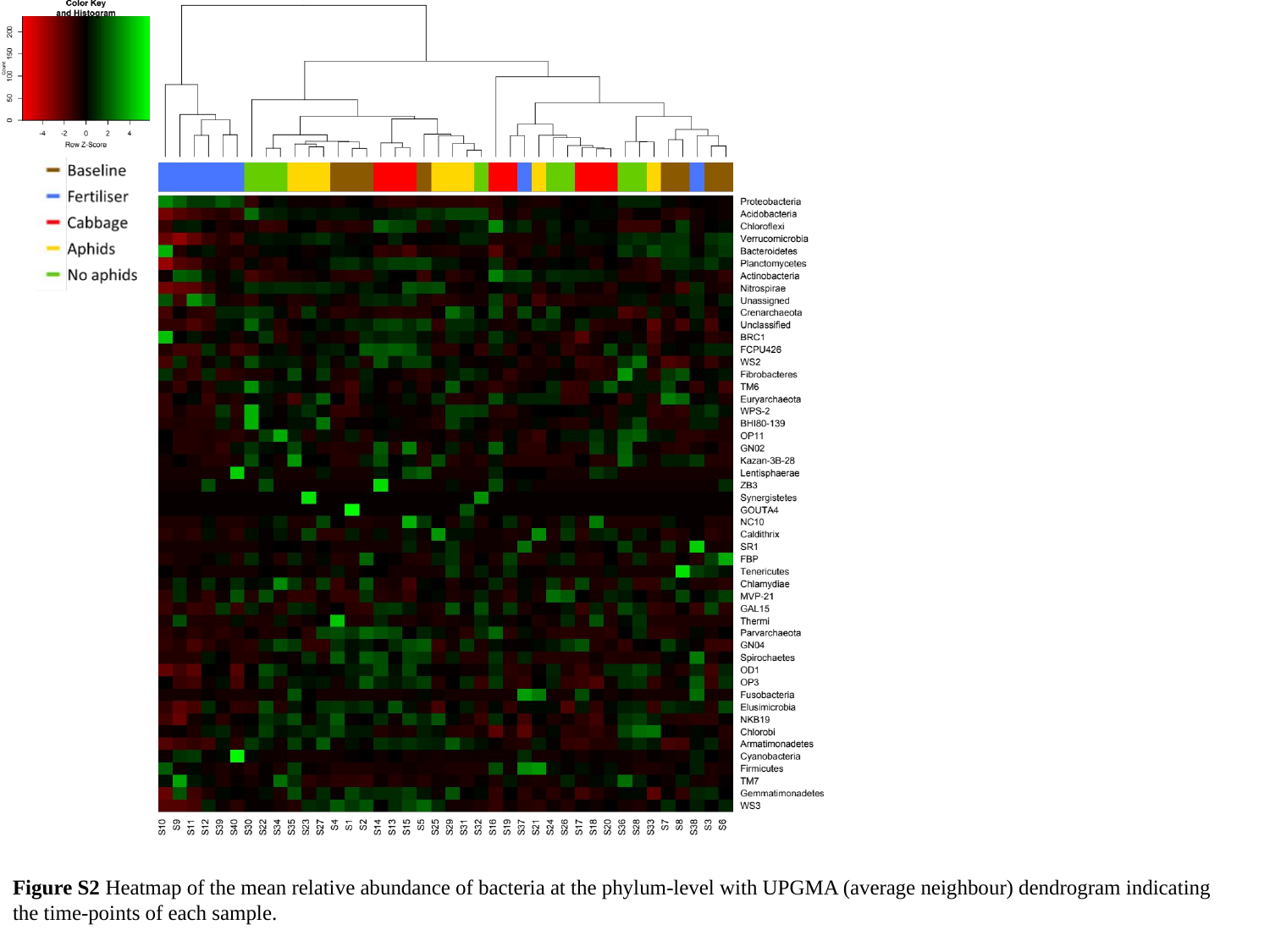

Figure S2 Heatmap of the mean relative abundance of bacteria at the phylum-level with UPGMA (average neighbour) dendrogram indicating the time-points of each sample.

## Slide 3
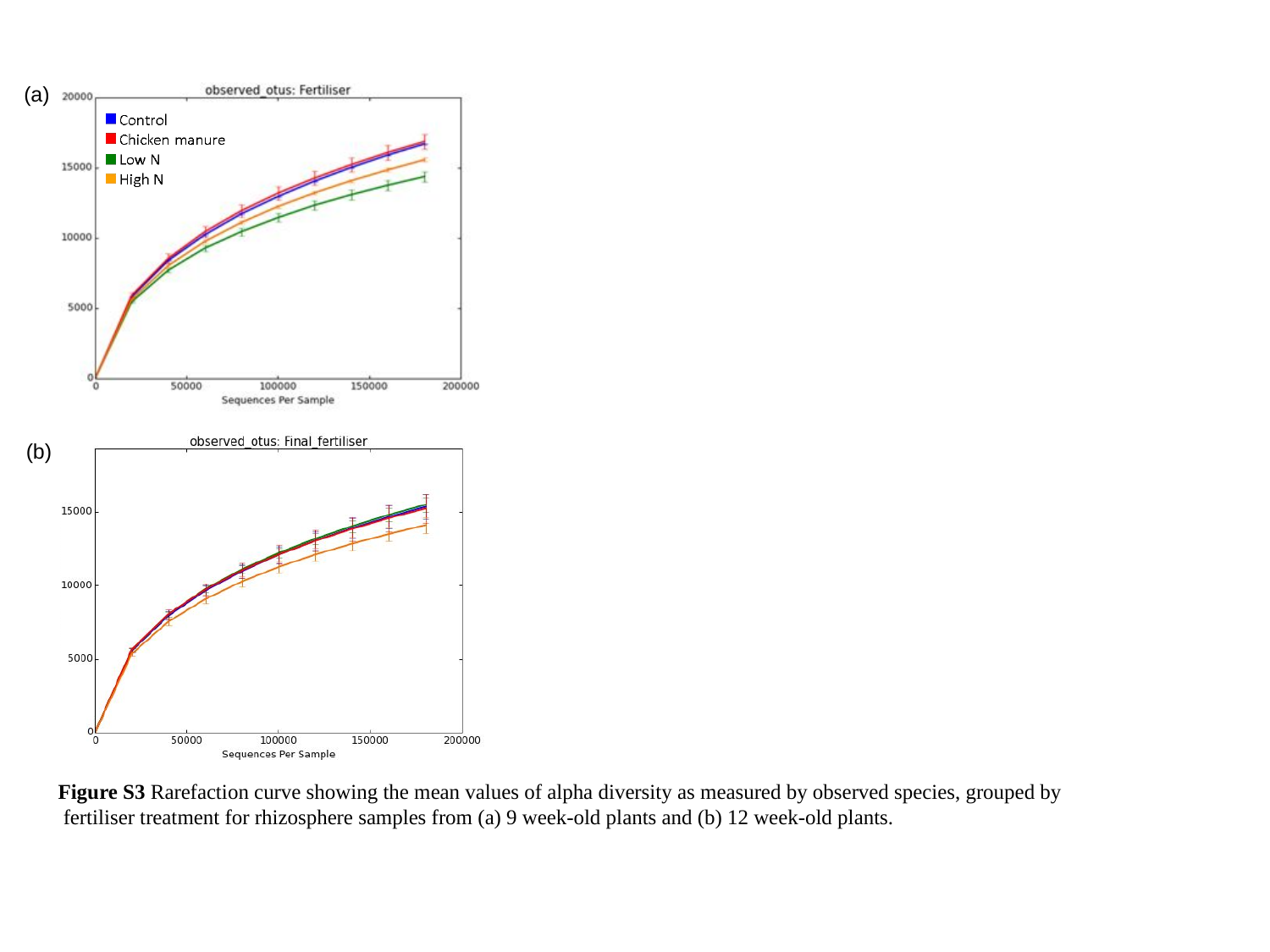

(a)
(b)
Figure S3 Rarefaction curve showing the mean values of alpha diversity as measured by observed species, grouped by
 fertiliser treatment for rhizosphere samples from (a) 9 week-old plants and (b) 12 week-old plants.

## Slide 4
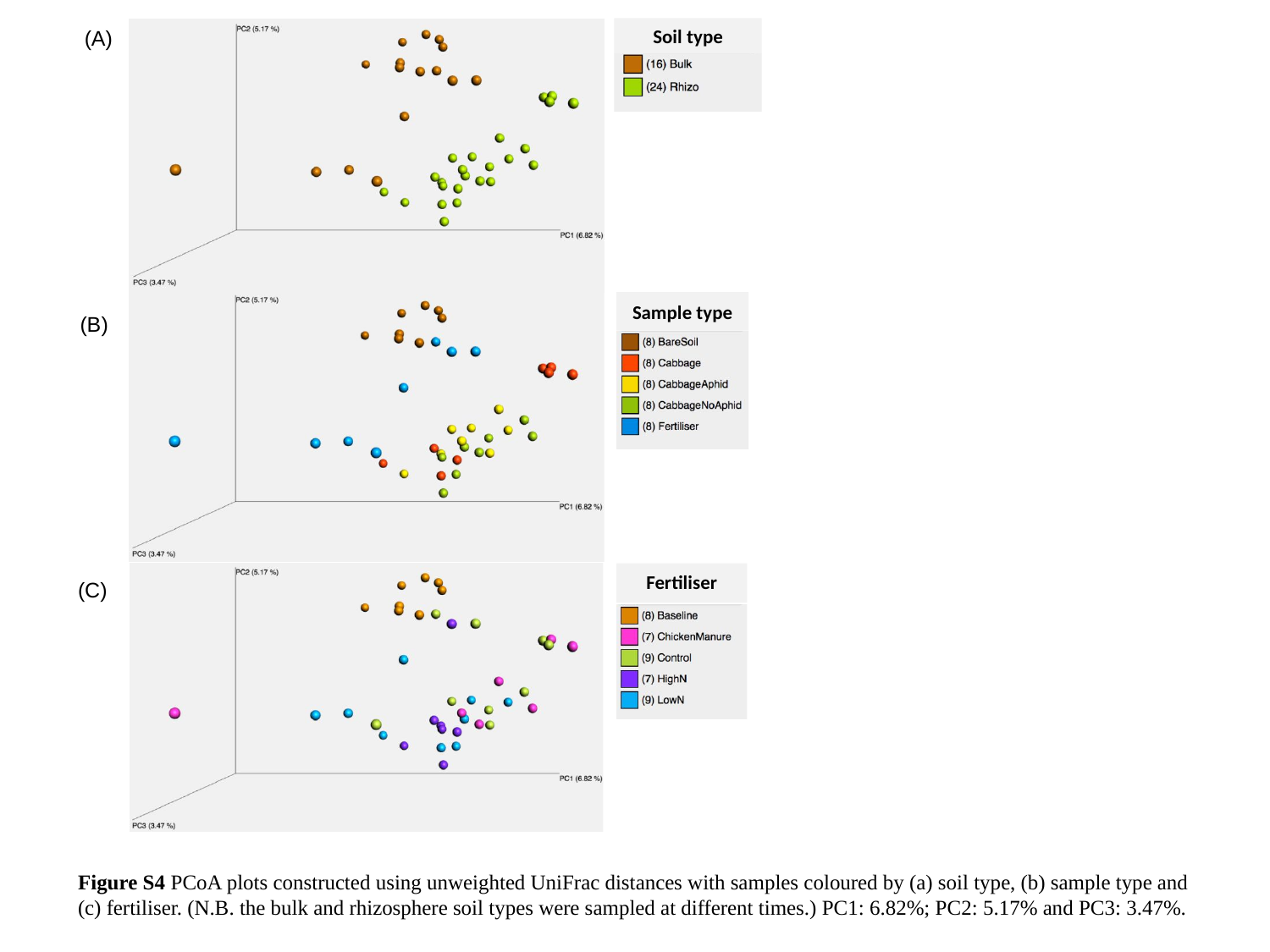

Soil type
(A)
(B)
Sample type
(C)
Fertiliser
Figure S4 PCoA plots constructed using unweighted UniFrac distances with samples coloured by (a) soil type, (b) sample type and (c) fertiliser. (N.B. the bulk and rhizosphere soil types were sampled at different times.) PC1: 6.82%; PC2: 5.17% and PC3: 3.47%.

## Slide 5
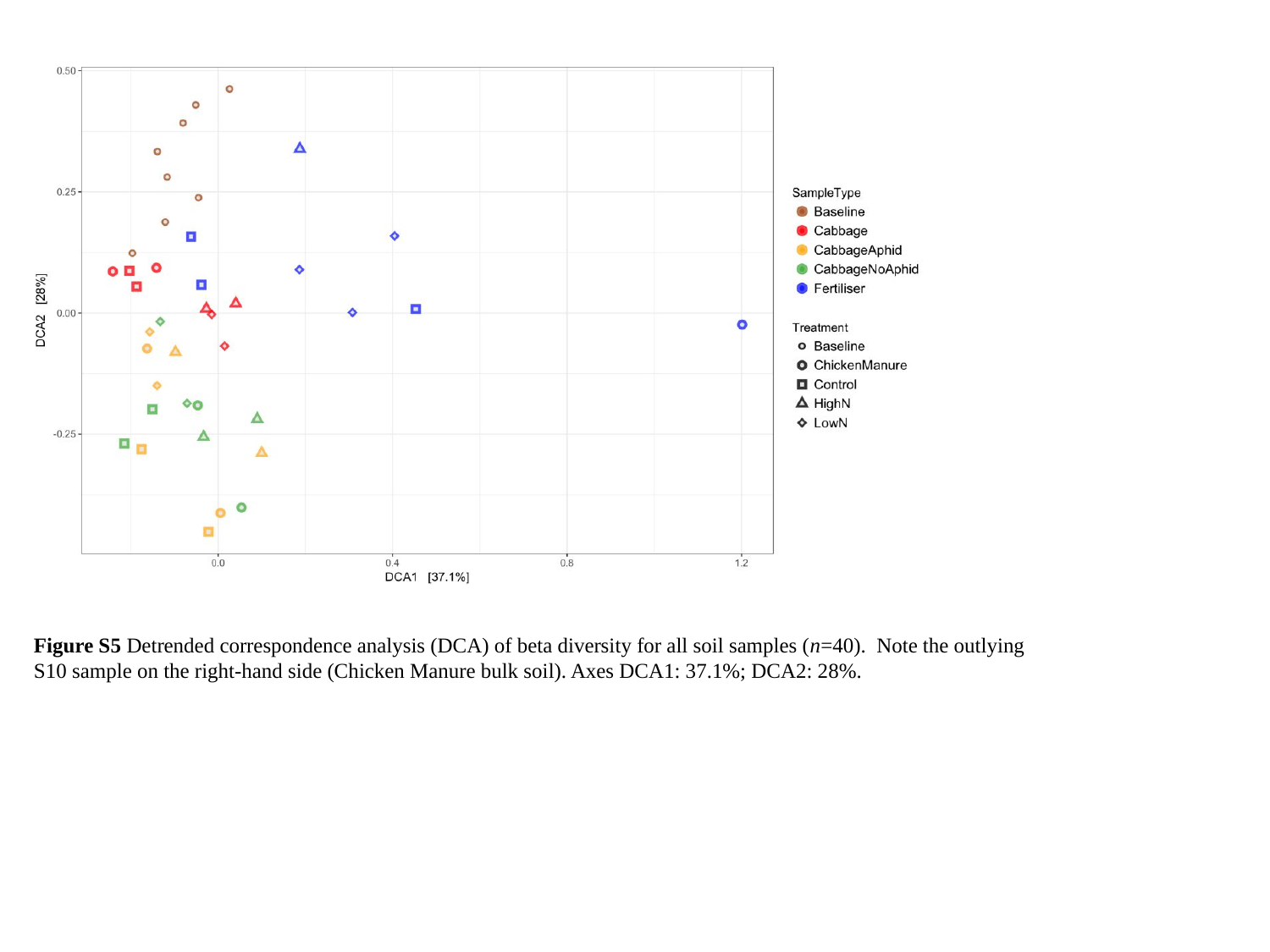

Figure S5 Detrended correspondence analysis (DCA) of beta diversity for all soil samples (n=40). Note the outlying S10 sample on the right-hand side (Chicken Manure bulk soil). Axes DCA1: 37.1%; DCA2: 28%.

## Slide 6
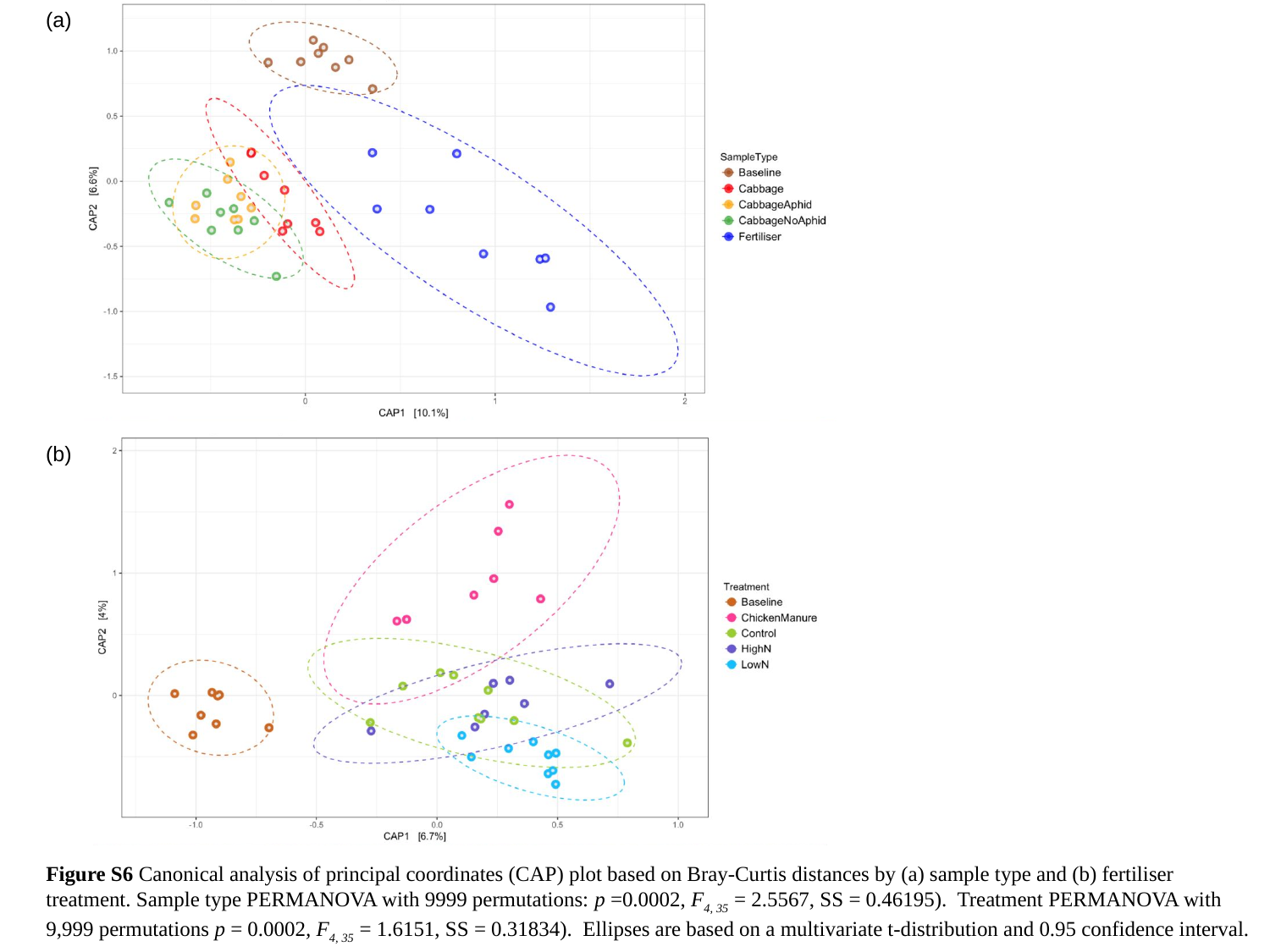

(a)
(b)
Figure S6 Canonical analysis of principal coordinates (CAP) plot based on Bray-Curtis distances by (a) sample type and (b) fertiliser treatment. Sample type PERMANOVA with 9999 permutations: p =0.0002, F4, 35 = 2.5567, SS = 0.46195). Treatment PERMANOVA with 9,999 permutations p = 0.0002, F4, 35 = 1.6151, SS = 0.31834). Ellipses are based on a multivariate t-distribution and 0.95 confidence interval.

## Slide 7
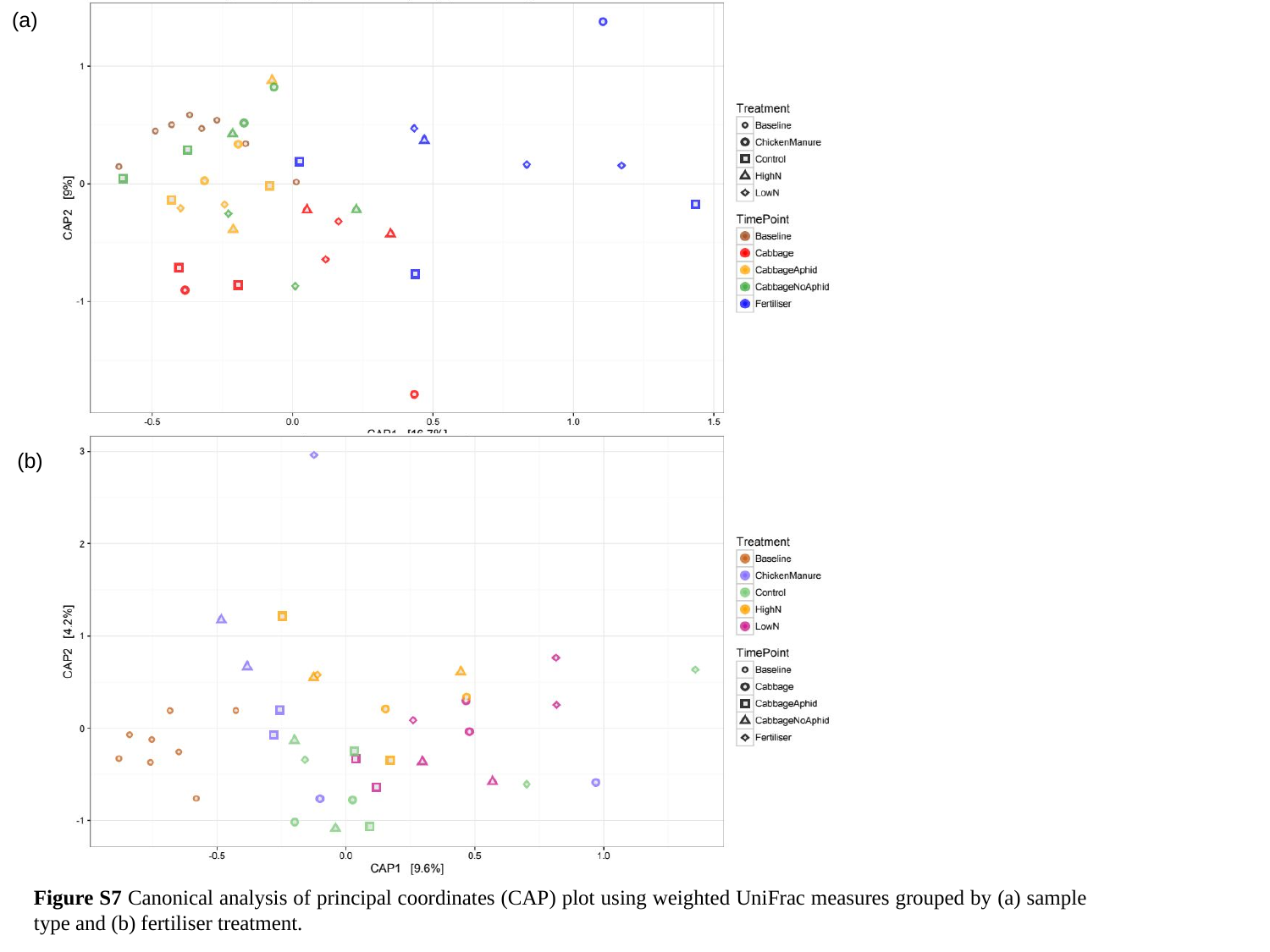

(a)
(b)
Figure S7 Canonical analysis of principal coordinates (CAP) plot using weighted UniFrac measures grouped by (a) sample type and (b) fertiliser treatment.

## Slide 8
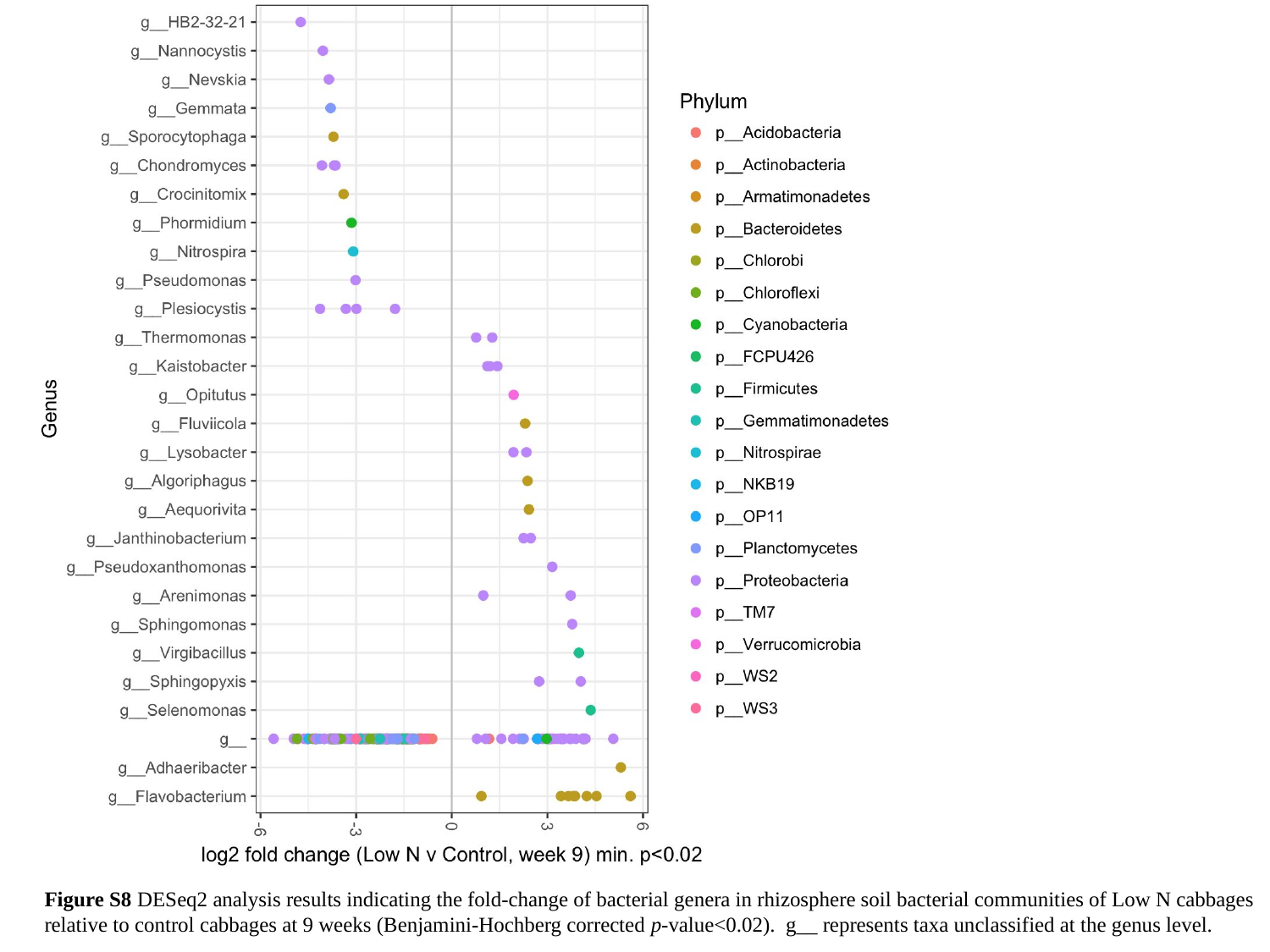

Figure S8 DESeq2 analysis results indicating the fold-change of bacterial genera in rhizosphere soil bacterial communities of Low N cabbages relative to control cabbages at 9 weeks (Benjamini-Hochberg corrected p-value<0.02). g__ represents taxa unclassified at the genus level.

## Slide 9
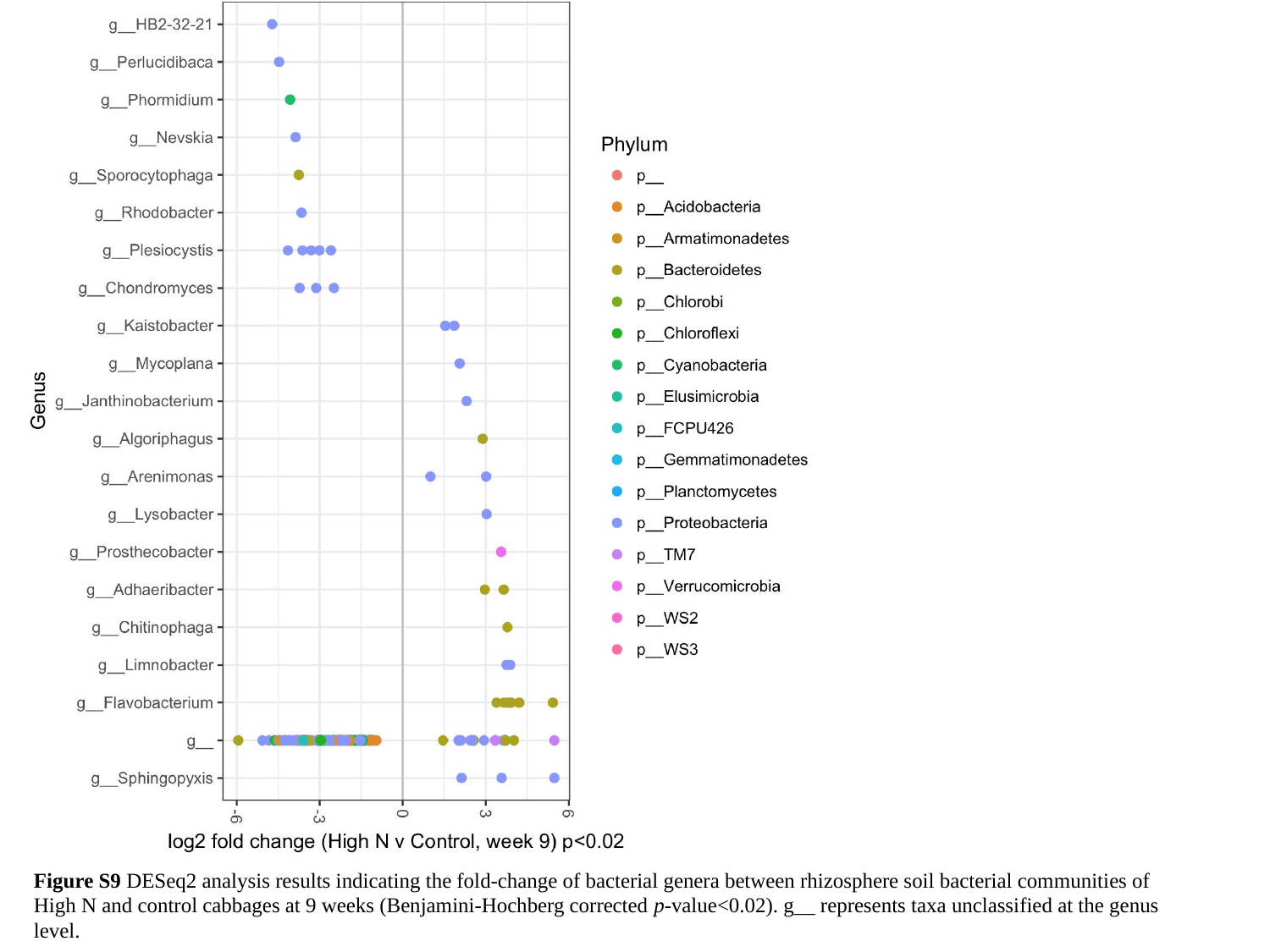

Figure S9 DESeq2 analysis results indicating the fold-change of bacterial genera between rhizosphere soil bacterial communities of High N and control cabbages at 9 weeks (Benjamini-Hochberg corrected p-value<0.02). g__ represents taxa unclassified at the genus level.

## Slide 10
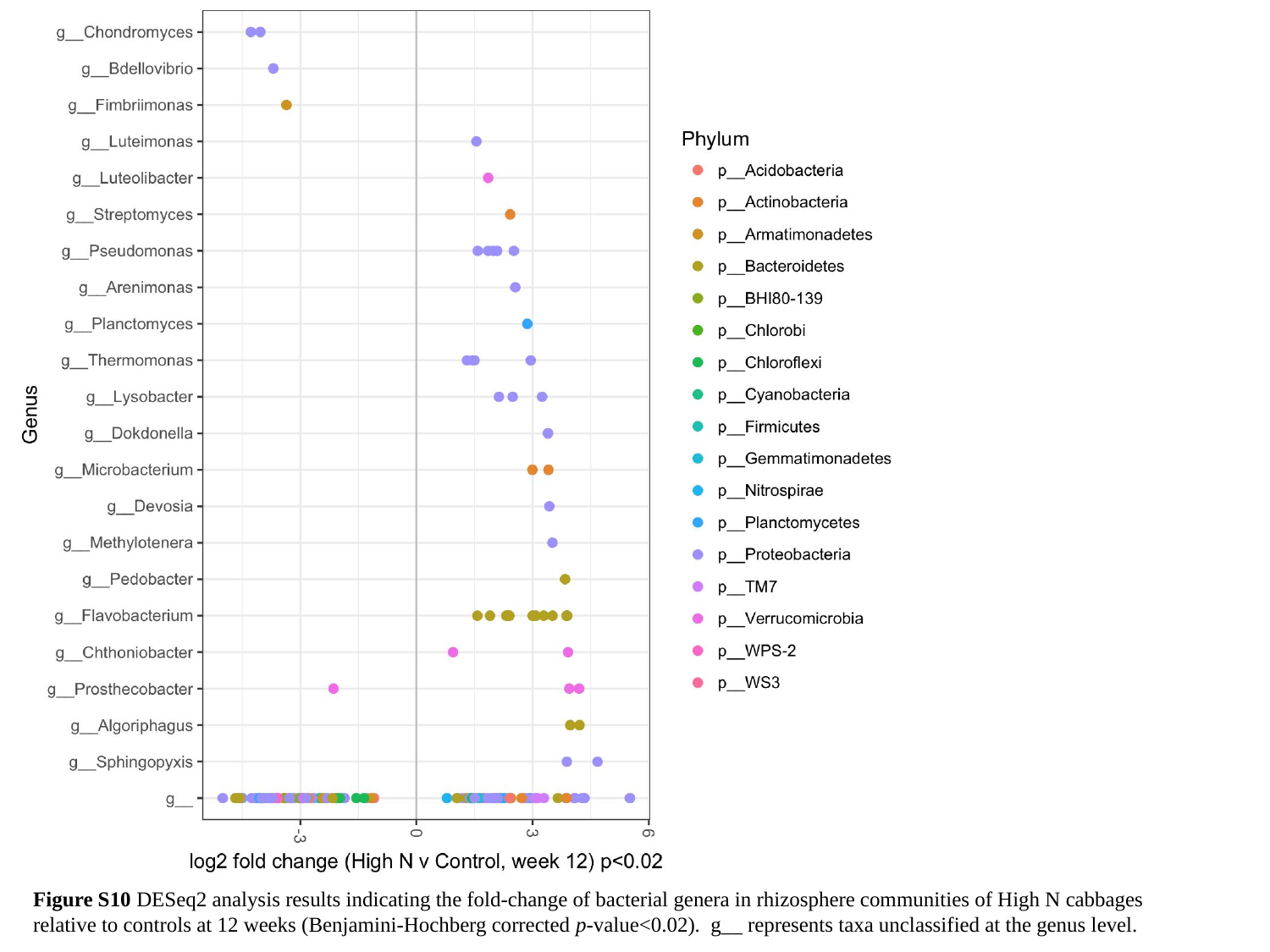

Figure S10 DESeq2 analysis results indicating the fold-change of bacterial genera in rhizosphere communities of High N cabbages relative to controls at 12 weeks (Benjamini-Hochberg corrected p-value<0.02). g__ represents taxa unclassified at the genus level.

## Slide 11
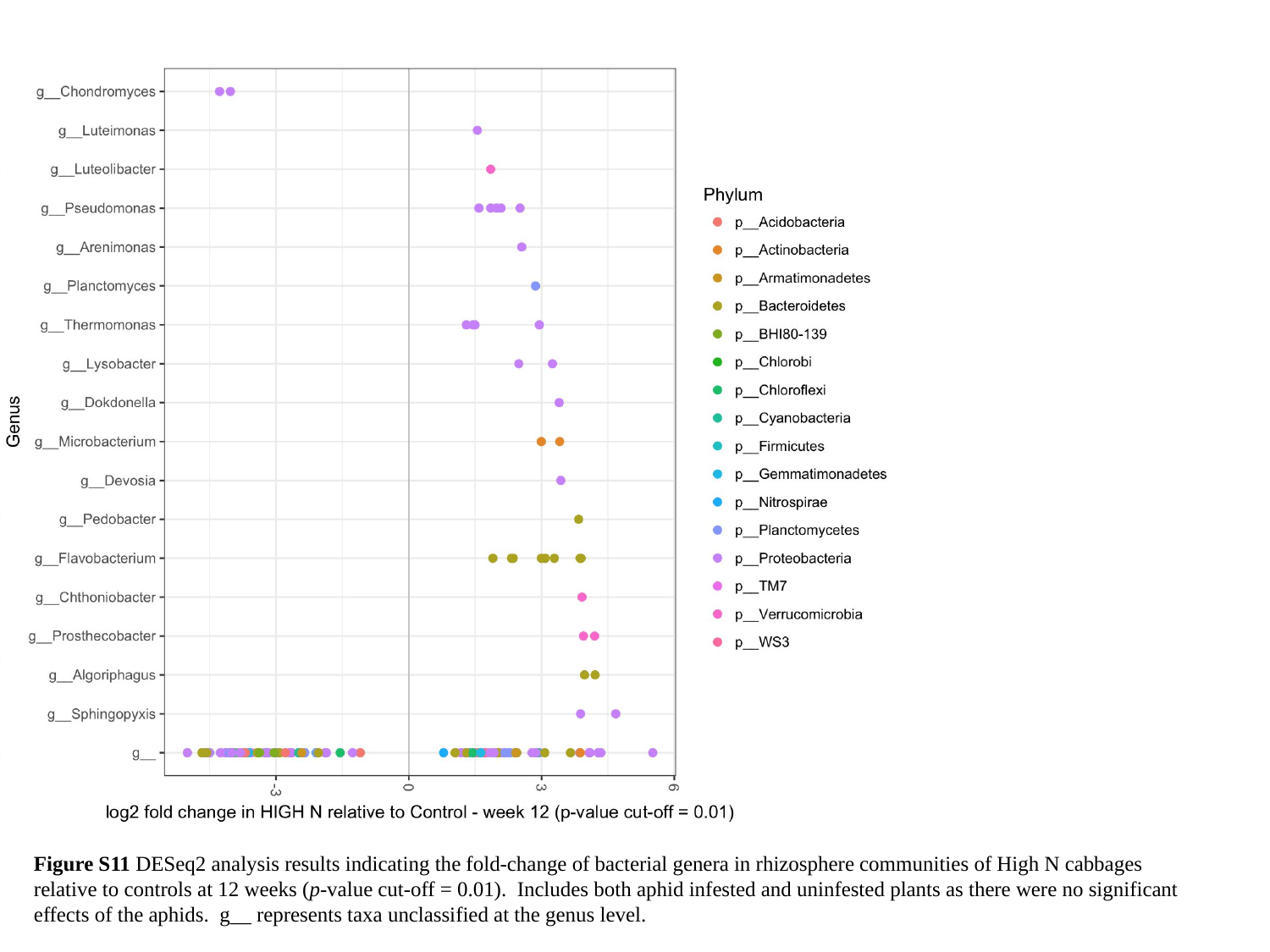

Figure S11 DESeq2 analysis results indicating the fold-change of bacterial genera in rhizosphere communities of High N cabbages relative to controls at 12 weeks (p-value cut-off = 0.01). Includes both aphid infested and uninfested plants as there were no significant effects of the aphids. g__ represents taxa unclassified at the genus level.

## Slide 12
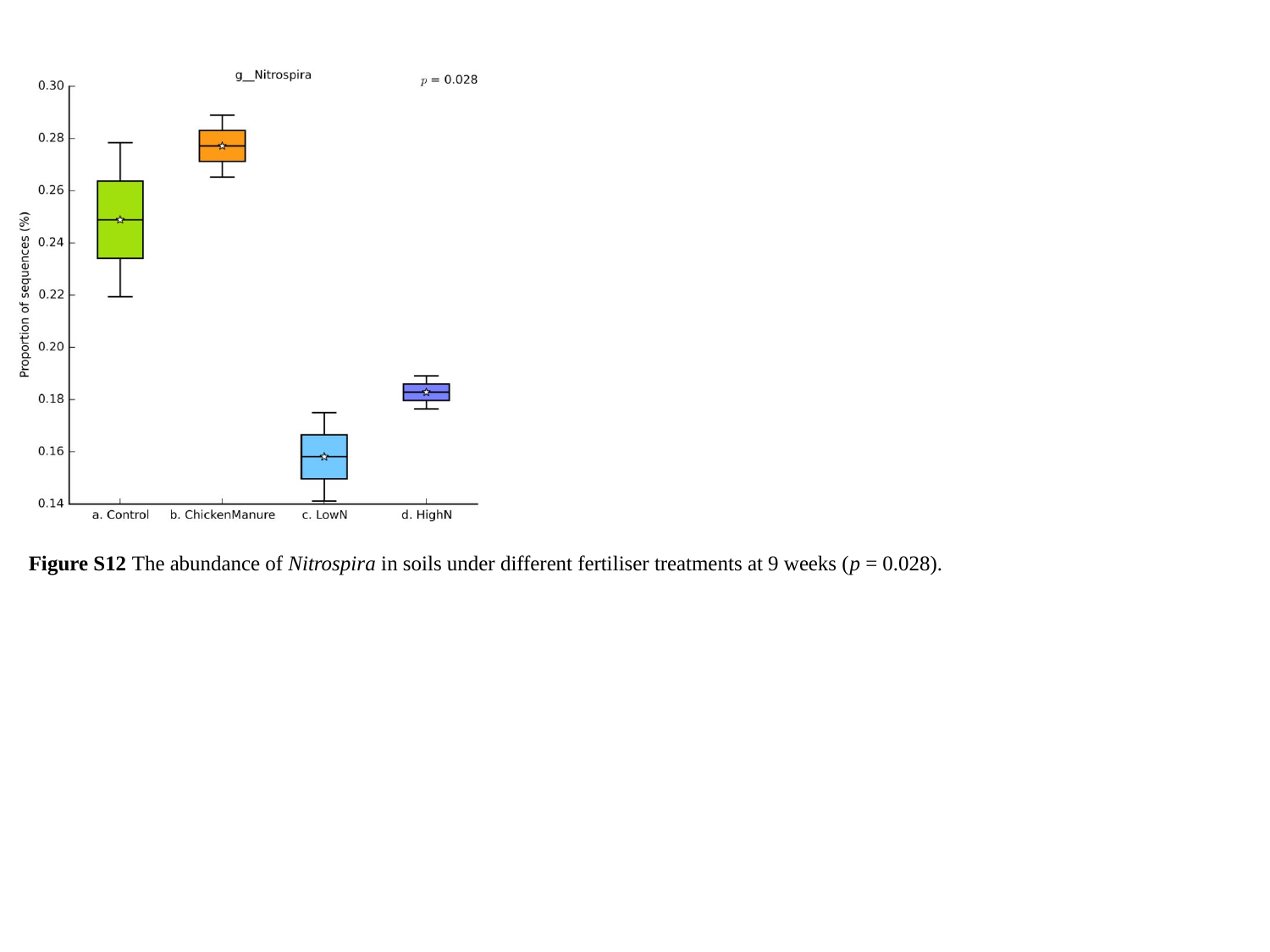

Figure S12 The abundance of Nitrospira in soils under different fertiliser treatments at 9 weeks (p = 0.028).

## Slide 13
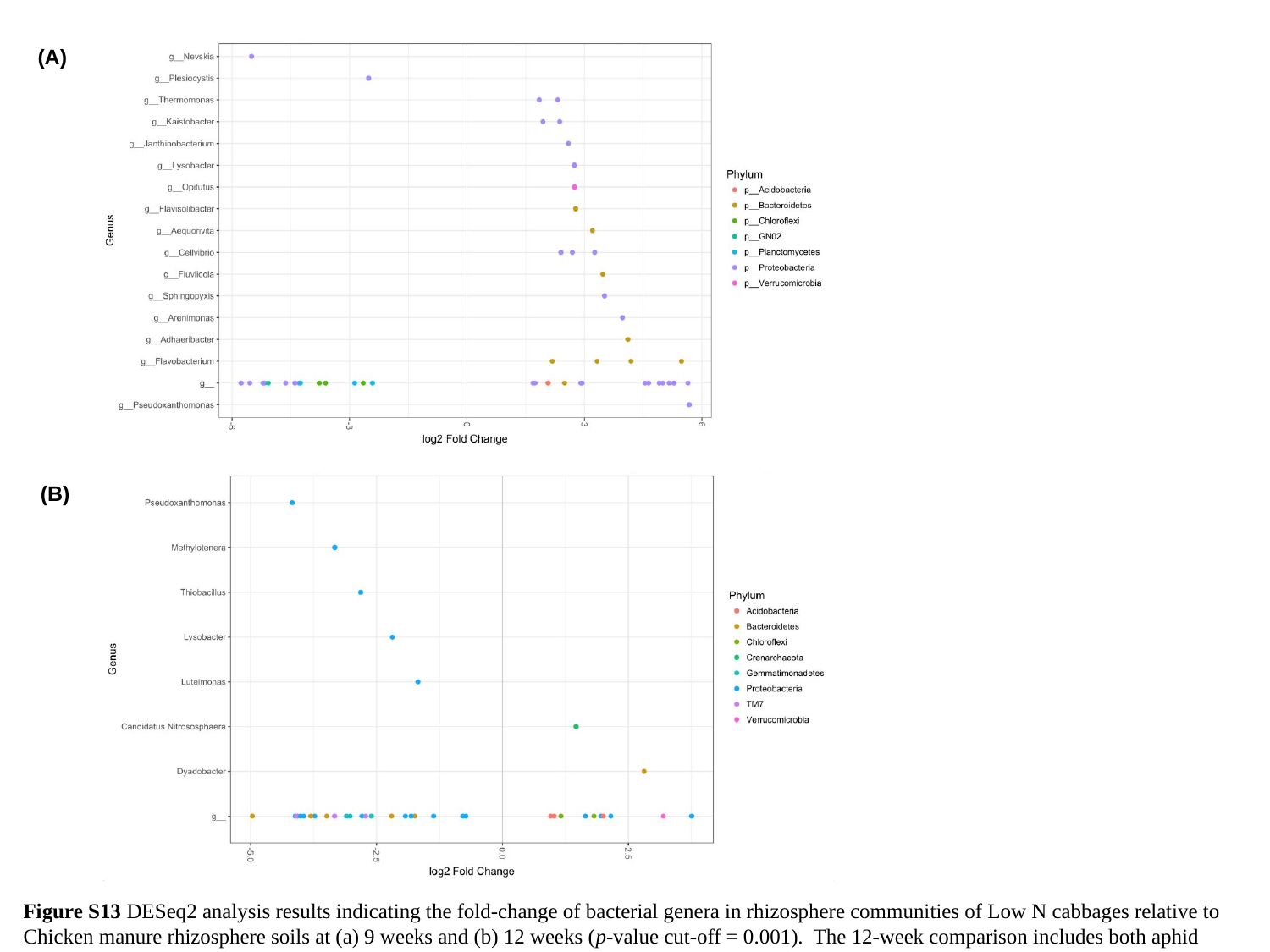

(A)
(B)
Figure S13 DESeq2 analysis results indicating the fold-change of bacterial genera in rhizosphere communities of Low N cabbages relative to Chicken manure rhizosphere soils at (a) 9 weeks and (b) 12 weeks (p-value cut-off = 0.001). The 12-week comparison includes both aphid infested and uninfested plants as there were no significant effects of the aphids. g__ represents taxa unclassified at the genus level.
