# Supplementary material for: Rhizosphere Bacterial Communities Differ According to Fertilizer Regimes and Cabbage (Brassica oleracea var. capitata L.) Harvest Time, but Not Aphid Herbivory
Source: Front Microbiol. 2018 Jul 23;9:1620. doi: 10.3389/fmicb.2018.01620 (PMC6064718; doi:10.3389/fmicb.2018.01620)
Supplement: Supplementary file 3 [file Data_Sheet_1.docx]

Supplementary Material

Rhizosphere Bacterial Communities Differ According to Fertilizer Regimes and Cabbage (*Brassica oleracea* var. L. *capitata* L.) Harvest Time, but Not Aphid Herbivory

Flora J. M. O’Brien^1, #,*^, Marc G. Dumont^1^, Jeremy S. Webb^1^ and Guy M. Poppy^1^

^1^ Biological Sciences, University of Southampton, Southampton, UK

^#^Present address: NIAB EMR, New Road, East Malling, ME19 6BJ, Kent, UK

*** Correspondence:** Dr. Flora O’Brien, flora.o’brien@emr.ac.uk

## Supplementary Methods

### 16S rRNA gene library preparation

The amplicon libraries for each of the 40 DNA samples were prepared through a two-step PCR amplification process to amplify the V4-region 16S rRNA gene for bacteria and archaea using the 16sv4 primer set with Illumina flowcell adapter sequences to enable for cluster formation (Caporaso, et al., 2012). The forward primer was 515F (5’-GTGCCAGCMGCCGCGGTAA-3’) and the reverse primer was 806R (5’-GGACTACHVGGGTWTCTAAT-3'). They amplify the 533–786 region of the *Escherichia coli* strain 83972 16S rRNA sequence (Greengenes accession no. prokMSA_id:470367). The primers also had 12bp Nextera barcode sequences (indices i7 and i5) to act as a unique barcode for each sample to enable multiplexing of all samples. The first step PCR used 5ng of each DNA sample, 0.5μl of each primer (10μM) and 10μl of 2x Kapa Hi Fi amplification mix, to give a total PCR reaction mix volume of 20μl. The cycling conditions were 95°C for 2 minutes, followed by 10 cycles of 98°C for 20 seconds, 65°C for 15 seconds, and 72°C for 30 seconds, with a final extension of 72°C for 5 minutes. The resulting amplicons were then cleaned using a magnetic bead capture kit (AMPure XP) at a ratio of 1:1 and re-suspended in 9μl. This was then used in the second PCR, after adding 0.5μl of each of the 8 base Nextera^®^ indices (i7 and i5) and Illumina sequencing adapters (both at 10μM) using the Nextera DNA kit (Illumina, UK), following the manufacturer’s instructions (Bartram, Lynch, Stearns, Moreno-Hagelsieb, & Neufeld, 2011). This second-step PCR again used 2x Kapa Hi Fi mix (10μl) under the same PCR conditions as before, but this time increasing it to 15 cycles. This second-step PCR used 8 forward and 5 reverse primers, used in unique combinations with index codes for each DNA sample to enable multiplex sequencing of all 40 samples. Next, the amplification products were again cleaned using 1:1 AMPure beads to remove very short library fragments, before the library normalization and pooling stages. The recovered amplicon pools were quantified and quality checked using the Qubit assay (Invitrogen) and Bioanalyzer (Agilent) DNA HS chip for peak distribution. The products were then pooled on an equimolar basis and purified using Prep (Sage Science) to select specifically for the amplicon band. The final concentration of the library pool was determined by quantitative PCR (qPCR) with the Illumina Library Quantification Kit (Kapa Biosciences) on a Roche Light Cycler LC480II, according to the manufacturer's instructions. The template DNA was denatured according to the Illumina MiSeq protocol, before being loaded at 7pM concentration, with the addition of 15% phage PhiX to increase the complexity of the amplicon library. The PhiX spike is required to correct for the extreme GC base bias present in 16S amplicon samples which can be as high as 69% for some bacteria and can result in errors in mean relative abundance estimates (de Muinck, Trosvik, Gilfillan, Hov, & Sundaram, 2017; Laursen, Dalgaard, & Bahl, 2017; Pinto & Raskin, 2012). This PhiX spike was later filtered out of the data set. The libraries were then pooled and sequenced in parallel on one flowcell of a MiSeq 2000 Illumina platform at 2x250bp paired-end sequencing with v2 chemistry.

### Bacterial community analysis

Alpha diversity metrics were calculated in QIIME using the *alpha_diversity.py* command with the results of *multiple_rarefactions.py* which implemented a series of subsampling (from 10 to 195,820 sequences per sample in increments of 20,000, with 10 iterations at each increment) to an even depth of 198,288 sequences. Chao1 gives an estimate of species richness; observed species (OTUs) metric gives a basic count of the number of unique OTUs per sample; and Faith’s PD is defined as the minimum total length of all the phylogenetic branches of a phylogenetic tree comprising all the taxa contained within a sample. Statistically significant differences between α-diversity metrics of different treatments or sample types were tested for using the *compare_alpha_diversity.py* script in QIIME which employs a nonparametric two-sample t-test with 999 Monte Carlo permutations and Bonferroni multiple test correction. A heatmap with a phylogenetic UPGMA dendrogram was made using the *heatmap2* function in the *gplots* library in R.

Beta diversity indices were calculated in QIIME from the rarefied OTU table using the *jackknifed_beta_diversity.py* script, to a maximum depth of 190, 000. Distance matrices constructed using unweighted UniFrac distance metrics were visualised in PCoA plots using the programme EMPEROR (Vázquez-Baeza, Pirrung, Gonzalez, & Knight, 2013). A permutation test for homogeneity of multivariate dispersions (PERMDISP) was used to test for multivariate homogeneity of dispersions for each of the groups that yielded significant PERMANOVA results. The null hypothesis for PERMDISP assumes that the within-group dispersion of samples is similar for all groups (Anderson & Walsh, 2013). A non-significant PERMDISP result indicates that any significant PERMANOVA results can be confidently assumed to be attributed to differences in their centroids (i.e. the central location of a group of samples within the distance matrix). If the PERMDISP result is significant, then it is possible that a significant PERMANOVA result was generated due to unequal variation in the dispersion of the communities, rather than compositional contrasts in their communities (Erwin, Pita, López-Legentil, & Turon, 2012).

Ordination enables the visualisation of large data sets by reducing their dimensions either using distance-based or eigenvector-based methods (Erb-Downward, et al., 2012). Unconstrained ordination can be used to determine the most appropriate type of constrained ordination to apply to a data set, whilst constrained ordination can be used to test hypotheses. DCA plots were produced using the *decorana* function in the *vegan* package in R. DCA plots can be used to infer the type of data (i.e. homogeneous or heteroskedastic), which in turn can be used to select appropriate ordination methods for hypothesis testing. Lepš and Šmilauer (2003) recommended that linear ordination methods should be used for data where the first DCA axis is less than 3, and if it is more than 4 units in length then unimodal methods are more appropriate. Our DCA plot had first axis <2, and so a CAP (constrained PCoA) ordination was subsequently selected as an appropriate method to identify linear relationships between bacterial communities of the different sampling groups.

**References**

Anderson, M. J., & Walsh, D. C. (2013). PERMANOVA, ANOSIM, and the Mantel test in the face of heterogeneous dispersions: What null hypothesis are you testing? *Ecological Monographs* *, 83*, 557-574.

Bartram, A. K., Lynch, M. D., Stearns, J. C., Moreno-Hagelsieb, G., & Neufeld, J. D. (2011). Generation of multimillion-sequence 16S rRNA gene libraries from complex microbial communities by assembling paired-end Illumina reads. *Applied and Environmental Microbiology* *, 77*, 3846-3852.

Caporaso, J. G., Lauber, C. L., Walters, W. A., Berg-Lyons, D., Huntley, J., Fierer, N., et al. (2012). Ultra-high-throughput microbial community analysis on the Illumina HiSeq and MiSeq platforms. *The ISME Journal* *, 6*, 621-1624.

de Muinck, E., Trosvik, P., Gilfillan, G., Hov, J., & Sundaram, A. (2017). A novel ultra high-throughput 16S rRNA gene amplicon sequencing library preparation method for the Illumina HiSeq platform. *Microbiome* *, 5*, 68.

Erb-Downward, J., Akha, A., Wang, J., Shen, N., He, B., Martinez, F., et al. (2012). Use of direct gradient analysis to uncover biological hypotheses in 16S survey data and beyond. *Scientific Reports* *, 2*, 774.

Erwin, P. M., Pita, L., López-Legentil, S., & Turon, X. (2012). Stability of Sponge-Associated Bacteria over Large Seasonal Shifts in Temperature and Irradiance. *Applied and Environmental Microbiology* *, 78*, 7358-7368.

Laursen, M., Dalgaard, M., & Bahl, M. (2017). Genomic GC-Content Affects the Accuracy of 16S rRNA Gene Sequencing Based Microbial Profiling due to PCR Bias. *Frontiers in Microbiology* *, 8*, 1934.

Lepš, J., & Šmilauer, P. (2003). *Multivariate analysis of ecological data using CANOCO.* Cambridge University Press.

Pinto, A., & Raskin, L. (2012). PCR biases distort bacterial and archaeal community structure in pyrosequencing datasets. *PloS One* *, 7*, e43093.

Vázquez-Baeza, Y., Pirrung, M., Gonzalez, A., & Knight, R. (2013). EMPeror: a tool for visualizing high-throughput microbial community data. *GigaScience* *, 2*, 1-4.
